# A Multiagent Summarization and Auto-Evaluation Framework for Medical Text: Development and Evaluation Study

**DOI:** 10.2196/75932

**Published:** 2025-12-16

**Authors:** Yuhao Chen, Bo Wen, Farhana Zulkernine

**Affiliations:** 1 School of Computing, Queen's University, 557 Goodwin Hall, Kingston, ON, K7L 2N8, Canada, 1 6138930999; 2 Hogarthian Technologies, New York, NY, United States

**Keywords:** large language model evaluation, unstructured medical data summarization, multi-agent network, summarization evaluation, LLM as a judge, LLM

## Abstract

**Background:**

Although large language models (LLMs) show great promise in processing medical text, they are prone to generating incorrect information, commonly referred to as hallucinations. These inaccuracies present a significant risk for clinical applications where precision is critical. Additionally, relying on human experts to review LLM-generated content to ensure accuracy is costly and time-consuming, which sets a barrier against large-scale deployment of LLMs in health care settings.

**Objective:**

The primary objective of this study was to develop an automatic artificial intelligence (AI) system capable of extracting structured information from unstructured medical data and using advanced reasoning techniques to support reliable clinical decision making. A key aspect of this objective is ensuring that the system incorporates self-verification mechanisms, enabling it to assess the accuracy and reliability of its own outputs. By integrating such mechanisms, we aim to enhance the system’s robustness, reduce reliance on human intervention, and improve the overall trustworthiness of AI-driven medical summarization and evaluation.

**Methods:**

The proposed framework comprises 2 layers: a summarization layer and an evaluation layer. The summarization layer uses Llama2-70B (Meta AI) and Mistral-7B (Mistral AI) models to generate concise summaries from unstructured medical data, focusing on tasks such as consumer health question summarization, biomedical answer summarization, and dialog summarization. The evaluation layer uses GPT-4-turbo (OpenAI) as a judge, leveraging pairwise comparison strategies and different prompt strategies to evaluate summaries across 4 dimensions: coherence, consistency, fluency, and relevance. To validate the framework, we compare the judgments generated by the LLM assistants in the evaluation layer with those provided by medical experts, offering valuable insights into the alignment and reliability of AI-driven evaluations within the medical domain. We also explore a way to handle disagreement among human experts and discuss our methodology in addressing diversity in human perspectives.

**Results:**

The study found variability in expert consensus, with average agreement rates of 19.2% among all experts and 54% among groups of 3 experts. GPT-4 (OpenAI) demonstrated alignment with expert judgments, achieving an average agreement rate of 83.06% with at least 1 expert and comparable performance in cross-validation tests. The enhanced guidance in prompt design (prompt-enhanced guidance) improved GPT-4’s alignment with expert evaluations compared with a baseline prompt, highlighting the importance of effective prompt engineering in auto-evaluation of summarization tasks. We also evaluated open-source LLMs, including Llama-3.3 (Meta AI) and Mixtral-Large (Mistral AI), and a domain-specific LLM, OpenBioLLM (Aaditya Ura), for comparison as LLM judges.

**Conclusions:**

This study highlights the potential of LLMs as reliable tools for unstructured medical data summarization and evaluation to reduce the dependency on human experts and also states the limitations. The proposed framework, multiagent summarization and auto-evaluation, demonstrates scalability and adaptability for clinical applications while addressing key challenges like hallucination and position bias.

## Introduction

In the era of big data, health care presents unique challenges for researchers, particularly due to the vast amount of unstructured data that can significantly impact patient outcomes [1]. A key challenge is extracting structured information from unstructured medical texts to facilitate search, analysis, and summarization for clinical decision-making [2]. Automatic systems often struggle with information extraction and summarization from medical data due to the length and noise in clinical texts [3], making accurate interpretation challenging.

The rapid advancement of artificial intelligence (AI) is transforming the health care sector with large language models (LLMs) like GPT-4 (OpenAI) [4], which demonstrate impressive capabilities across a range of medical tasks [5-9]. For instance, older models like Med-PaLM (Google AI) and, subsequently, Med-PaLM 2 (Google AI), achieved 67.2% and 86.5%, respectively, on medical question text summarization tasks with US Medical Licensing Examination–style questions. Newer LLMs like GPT-4, on the other hand, achieved an impressive 90% accuracy on US Medical Licensing Examination questions, outperforming human test-takers [5]. GPT-4 also ranked within the top 10% of all participants on German medical licensing exams among medical students [6]. Other models, such as Gemini (Google) [10,11] and OpenBioLLM (Aaditya Ura) [12,13], have also shown robust performance in analyzing medical data. Such results highlight LLMs’ potential to support medical education and examination systems and aid health care practitioners in evidence-based decision support. However, despite these promising developments, LLM applications in health care remain primarily experimental. One of the critical limitations is hallucination, where LLMs generate content that is incoherent or factually incorrect [14,15]. This issue poses significant risks in clinical settings, as hallucinated outputs can lead to erroneous diagnoses, inappropriate treatments, mental distress, and reduced trust in AI-driven systems. Conventional similarity-based evaluation metrics, such as ROUGE [16] and BERTScore [17], are not able to detect hallucinations. Moreover, these similarity-based automatic evaluation metrics require expert-generated gold standard summaries. Compared with other domains, medical text annotation requires expertise from specialized health care professionals, making it significantly more expensive. With the success of the advancements of LLM models, there has been a growing interest in developing human-free automatic evaluation frameworks that can leverage LLM’s capabilities.

This research is motivated by the growing need for accurate medical summaries, which are critical for clinical reviews, assessments for insurance, and audit processes. The vast volume of medical data generated every day, such as patient records and laboratory results, highlights the demand for advanced automatic summarization technologies. Traditional methods, such as manual summarization or rule-based systems, often struggle to efficiently extract relevant information, leading to delays or inaccuracies in clinical workflows.

To address these challenges, this study aims to answer the following primary research question: “How to build an AI system that can self-evaluate its performance across various medical summarization tasks?” It specifically explores the following key aspects:

1. How well can LLMs generate summaries from medical domain-specific data?
2. How do prompt design and position bias influence the reliability of LLM-based evaluations, and how to enhance the reliability?
3. How do LLMs perform compared with human experts in evaluating the generated summaries in terms of reliability?

To answer the above questions, this study focuses on developing an automatic medical question, dialog, and descriptive text summarization and evaluation system, multiagent summarization and auto-evaluation (MASA), which serves as a case study to explore how LLMs can improve the extraction of essential insights from complex, unstructured medical data. The key objectives include ensuring medical accuracy, relevance, and proper evaluation of the generated summaries, as a foundational step toward broadening AI applications in health care data processing. Notably, this work presents one of the first multiagent frameworks that integrates open-source summarization models (Llama [Meta AI] and Mistral [Mistral AI]) with an LLM as a judge (GPT-4), and evaluates its performance. We study its agreement with human experts across diverse medical summarization tasks using 5 distinct datasets (MEDIQA-QS, MeQSum, MEDIQA-ANS, MEDIQA-MAS, and MedDialog-EN). We also assess some of the newer LLMs, such as Llama 3 (Meta AI), Mixtral-Large (Mistral), and OpenBioLLM (Aaditya Ura) to provide a brief comparison of their performance as evaluators.

## Methods

### Overview

**Figure 1.**
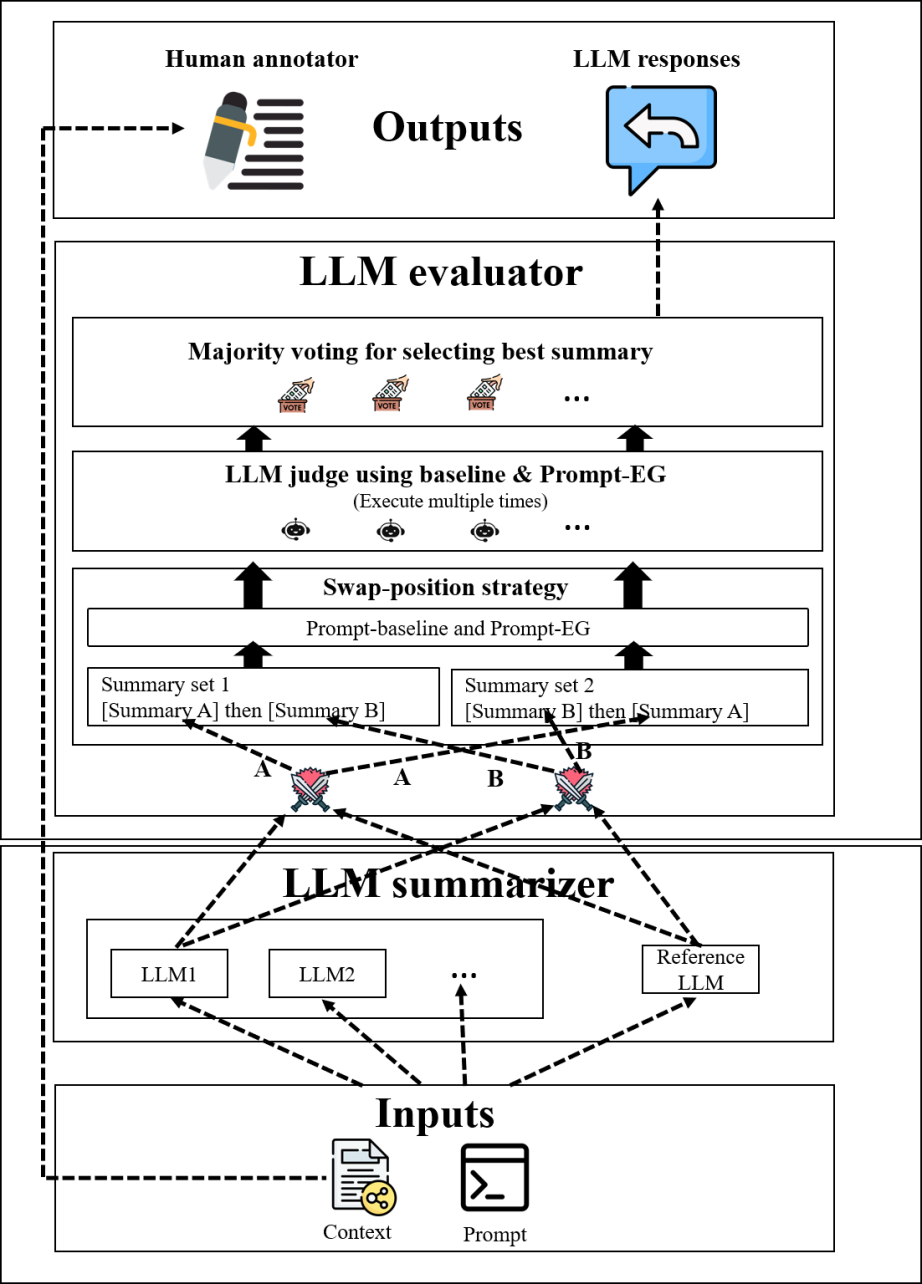
The proposed multi-agent LLM framework with two layers performing summarization and then evaluation. EG: enhanced guidance; LLM: large language model.

The overall architecture of our proposed system is illustrated in Figure 1, which represents a multilayer LLM agent network composed of 2 distinct layers. It is inspired by IBM’s SOLOMON (System for Optimizing Language Outputs through Multi-agent Oversight Networks) framework [18]. The 2-layer approach implements a comprehensive processing pipeline that distills and validates medical information, contributing to more effective and timely health care decision support.

### Summarization Layer

The network begins with a summarization layer, where LLM summarizers extract key information from unstructured data sources such as patient inquiries, doctor-patient conversations, and detailed question answers. This initial layer focuses on transforming lengthy and disorganized medical content into concise summaries that retain essential information for further analysis.

### Evaluation Layer

#### Overview

The second layer, the evaluation layer, deploys LLM judges to assess the quality of the summaries generated by the summarization layer. Rather than using judges to grade each LLM’s summaries sequentially, we implement a pairwise comparison strategy where LLM judges compare and evaluate a pair of summaries to determine the superior one. This approach enables judges to focus on the subtle differences between the 2 responses, highlighting strengths and weaknesses that might otherwise be overlooked, thereby enhancing the overall precision of the evaluation process.

We adopt the *Referenced Condorcet Assessment (RCA*) [19] approach to optimize the pairwise evaluation process. Instead of comparing every model’s output against all others, RCA designates one “reference LLM” summarizer in the summarization layer and compares each model’s output against this reference. In a general pairwise evaluation, $\frac{n(n-1)}{2}$ comparisons are required for *n* LLM summarizers. With RCA, we significantly reduce the required number of comparisons to *n-1*, optimizing computational efficiency while maintaining accuracy in identifying high-quality summaries. By establishing a consistent reference point, RCA ensures consistent evaluation across different datasets. This approach provides reproducibility and improves the overall efficiency of the AI-evaluation layer.

#### Confidence and Bias in Evaluation

We implement a *majority voting mechanism* using multiple independent runs of the same LLM evaluator to enhance confidence in the decision. A final decision is reached when more than half of the evaluators agree; otherwise, the result is marked as a tie.

To address *position bias*, where the order of input summaries affects the evaluation outcome, we use a *swap-position strategy*. This involves evaluating each summary pair twice, the second time with reversed ordering. A summary is declared the winner only if it is preferred in both evaluations; otherwise, the outcome is considered a tie.

The name of the LLM summarizer is hidden in the evaluation process to promote anonymous and fair judgment. In the final step, when comparing outcomes with human experts, the same setting is used for human evaluators to ensure anonymity, fairness, and mitigate bias. Specifically, the source of each summary is anonymized, and the presentation order of summaries is randomized to minimize potential ordering and source biases.

### LLM Model Selection

We select 2 open-source LLMs to generate summaries in the summarization layer. At the time of this study, Llama2 was the state-of-the-art (SOTA) open-source model, making the *Llama2-70B-chat-hf version* an ideal choice [20]. We also selected *Mistral-7B-Instruct-v0.1*, which was optimized for faster inference and demonstrated superior performance, surpassing the larger Llama2-13B model in some tasks [21]. By choosing 2 different LLMs, we benefit from both Mistral’s strong performance and architectural diversity and reduce the risk of model-specific biases. We select GPT-3.5 (turbo) as the reference LLM for RCA because, compared with LLMs of similar generation in the Claude and Gemini families, GPT-3.5 (turbo) has been significantly more widely adopted in previous research and consistently used as a strong baseline in a variety of research [6,8].

For evaluation, we primarily selected the paid version of GPT-4 (turbo) as a judge, which was the SOTA LLM reasoning model at the time of this study [4]. Subsequently, we incorporated additional open source LLM evaluators, including Llama-3.3-70B-Instruct, Mixtral-large, and OpenBioLLM-70B. OpenBioLLM-70B [12] is a model trained on large-scale biomedical corpora. These models were used to examine variation in automated judgments and to assess their alignment with expert human evaluations. A summary of all the LLM models used in this study is illustrated in Table 1.

**Table 1.** Model specifications and generation settings for open-source and proprietary large language models used as summarizers, reference models, and evaluators.

Model descriptions	Llama2-70B-chat-hf	Mistral-7B-Instruct-v0.1	GPT-3.5 (turbo)	GPT-4 (turbo)	Llama-3.3-70B-Instruct	Mixtral-large	OpenBioLLM-70B
Parameters	70B	7B	—^a^	—	70B	123B	70B
Type	Sum^b^	Sum	Ref^c^	Eval^d^	Eval	Eval	Eval
Temperature	0.7	0.7	0.7	0	0	0	0
Top_p	1	1	1	0.1	0.1	0.1	0.1
Max_new_tokens	512	512	512	612	612	612	612

a Not available.

b Sum: summarizers.

c Ref: reference models.

d Eval: evaluators.

Furthermore, 2 key aspects of the proposed architecture and methodology include the adaptability to use any LLM in each of the layers and the robust information processing framework.

### Data Sources

We do not fine-tune any of the LLMs on the selected datasets. We validate our system for three distinct summarization tasks with different benchmark datasets presenting diverse challenges as described below. These datasets are commonly used in medical text analysis tasks [22].

#### Task 1: Consumer Health Question Summarization

Patients often ask detailed and complex health-related questions. The primary difficulty lies in reducing redundancy while preserving critical medical details. This challenge has been explored in benchmark datasets such as MEDIQA-QS [23] and MeQSum [24], both of which contain consumer health questions with gold-standard summaries crafted by medical experts. Consumer health question summarization can help to analyze service quality, consumers’ needs and concerns, and thereby improve support quality.

*MEDIQA-QS* comprises deidentified consumer health questions sourced from the US National Library of Medicine. The *MeQSum* dataset contains 1000 consumer health questions to facilitate natural language processing (NLP) research specifically for the medical field.

#### Task 2: Biomedical Answer Summarization

Answers to biomedical queries from multiple sources or services must be aggregated to produce a coherent summary. This task poses 2 main challenges: first, extracting and generating relevant nonredundant information based on the input question, and second, ensuring consistency across diverse information sources. In this study, we focus on addressing the second challenge by aggregating and summarizing multiple relevant responses to a single medical query. We conduct our analysis using MEDIQA-ANS [25] and MEDIQA-MAS [23] datasets.

*MEDIQA-MAS* consists of collections of responses to specific health-related queries, along with summaries created by experts that integrate information from multiple answers into a single coherent summary. *MEDIQA-ANS* contains health questions asked by consumers, their corresponding answers, and expert-created summaries. The dataset is sourced from reliable organizations such as MedlinePlus and derived from questions submitted to the National Library of Medicine’s CHiQA system.

#### Task 3: Dialog Summarization

Online medical advising uses interactive chat or voice to learn customer needs and provides appropriate responses based on expert knowledge. Key challenges in processing the data include handling informal expressions, identifying and filtering relevant medical information, and maintaining the temporal context of symptoms and events. Dialogue summarization is also important to learn about previous interactions with the same consumer to provide informed responses. We perform the task of producing a summary of a patient’s medical condition from a dialogue between the patient and the doctor and validate our approach using the MedDialog-EN [26] dataset.

*MedDialog-EN* is an extensive collection of real-life anonymized medical consultations between patients and physicians. It covers a wide range of medical inquiries and responses, demonstrating a doctor’s approach to various health problems.

We confirm that Tasks 1 and 2 datasets are published under the Creative Commons Attribution 4.0 International License (CC BY 4.0), which permits unrestricted use, distribution, and reproduction in any medium, provided the original work is properly cited. For Task 3, we have obtained explicit written permission from the original authors to reuse their content in this study, including its integration via the OpenAI application programming interface.

### Prompt Design

We explore the impact of prompt design on the performance of LLMs across both layers in our framework, performing summarization and evaluation.

#### Prompt Design for Summarizer

For effective benchmarking and comparison with existing research for the summarizer, we leverage the established prompt templates proposed by Jahan et al [27] as a foundation. Examples of prompts are given in Multimedia Appendix 1. The designed constant prompt for all the summarizer LLMs serves as a control variable and allows for the analysis of different prompt design approaches for the evaluator, ensuring that any variations in performance are attributed to the evaluator’s prompt design rather than changes in the summarizer’s prompt.

#### Prompt Design for Evaluator

##### Overview

For the evaluator, we introduce 2 custom prompt design approaches and systematically assess how variations in evaluator prompts influence the quality and reliability of the evaluation process. By comparing the prompts and outcomes, we aim to identify optimal strategies for leveraging LLMs as evaluators in summarization evaluation tasks.

##### Prompt Baseline

Our initial prompt design (Multimedia Appendix 1) for evaluator LLMs is inspired by the work of Zheng et al [28], which pioneers the use of LLM as a judge in evaluating the performance of other LLMs in primarily question-answering tasks. We adapt their approach for summarization tasks by enhancing the prompt to ensure that GPT-4 focuses on 4 key evaluation criteria commonly used by humans: *coherence, consistency, fluency,* and *relevance*. Additionally, we include statements in defining the baseline prompt for the evaluator LLMs to mitigate positional bias so that the order of summaries does not influence the LLM’s judgment.

##### Prompt-Enhanced Guidance

Based on the observation of the performance of the baseline prompt, several aspects of the baseline prompt require improvements to make the prompt more focused and less ambiguous. In *prompt-baseline*, terms used to define the evaluation criteria, namely “coherence,” “consistency,” “fluency,” and “relevance,” lack clear operational definitions and lead to inconsistencies in evaluation. We propose *prompt-enhanced guidance* (EG), a significantly more comprehensive prompt template that provides clearer and more detailed instructions. The update includes detailed descriptions of the 4 key evaluation criteria and integrates a chain-of-thought [29] guideline, which guides GPT-4 through a structured, step-by-step evaluation process. Prompt-EG also introduces several new instructions for: (1) balancing brevity and detail to facilitate the selection of a high-quality summary; (2) detecting hallucinations to remove improper content from the generated summary, and (3) ensuring factual consistency, which is the most critical evaluation factor in the medical domain. The prompt template is explained in Multimedia Appendix 1.

### Experiments

To evaluate the efficacy of our 2-layer framework, we perform rigorous experimentation on 3 summarization tasks using unstructured medical questions, dialogues, and long descriptive question answers. Examples of a few data samples used in the study are illustrated in Multimedia Appendix 2.

#### Evaluation by LLM

Each LLM evaluator compares a pair of anonymized summaries generated by 2 LLM assistants (A and B with no model names), and generates A, B, or a tie as the outcome. The same evaluation is conducted by human medical experts given the same summary data and evaluation criteria, and interrater agreements are analyzed.

#### Evaluation by Human Experts

A panel of 4 medical experts independently evaluated randomly selected 250 sample summary pairs, indicating their preferences based on clinical accuracy, completeness, and clarity. All experts received a standardized written guideline outlining the evaluation criteria, namely coherence, consistency, fluency, and relevance. For each pair, experts were instructed to select the better summary or mark them as a “tie” when both pairs were judged to be of equivalent quality. Experts were also allowed to abstain from evaluating specific pairs if the content was ambiguous, outside their area of expertise, or otherwise unsuitable for judgment. As a result, the final sample size included 246 evaluated summary pairs (47, 49, 50, 50, and 50 from the five datasets, respectively). All expert annotators participated voluntarily and did not receive financial compensation. Due to time and cost constraints, we could only recruit 4 human experts in this study. In addition to expert evaluation, 2 human annotators without formal medical education were recruited to assess the same summary pairs. These nonexpert annotators collaborated to reach a joint decision for each pair, using the same evaluation guidelines as the experts. This comparison allowed us to examine potential alignment and discrepancies between expert and nonexpert judgments. To assess the performance of the LLM judge, we compared the agreement rate (AR) between human annotators and the LLM judge for the summaries produced by LLaMA-2-70B and GPT-3.5.

#### Validation Criteria

We designed a comprehensive validation process that assesses the following capabilities of our 2-layer framework.

First, LLM-based summarization and evaluation were conducted by (1) evaluating the quality of the generated summaries, and (2) evaluating the impact of prompt design on evaluation consistency.

Second, inaccuracy detection (remove if not do) and bias mitigation were assessed by (1) inspecting task-specific trends and position bias; (2) analyzing the strengths and weaknesses of the framework’s hallucination detection; (3) analyzing agreement patterns between the LLM evaluator and human experts when selecting the better summary from a pair; and (4) investigating causes of disagreement by examining alignment among human experts, nonexperts, and LLMs across summarization tasks with varying data complexity.

Finally, the applicability of the framework in real life was assessed by (1) analyzing agreement patterns between the LLM evaluator and human experts when selecting the better summary from a pair, and (2) investigating causes of disagreement by examining alignment among human experts, nonexperts, and LLMs across summarization tasks with varying data complexity.

Specific experimental setups are reported with the outcomes under the results section using a set of evaluation metrics as discussed below.

#### Evaluation Metrics

##### AR

We compute AR to show how often judges agree by selecting the same summary as the preferred one or a tie in pairwise evaluations. We compute AR between LLM and human judges as well as among different subgroups of judges, as human judges tend to disagree due to subjective opinions. AR reflects alignment in judgment and is mainly used to compare the consistency of LLM versus human preferences. A higher AR indicates stronger agreement. Formal definitions are provided in Multimedia Appendix 3.

##### Cohen κ Scores

We also compute pairwise Cohen κ scores for each dataset to assess interrater reliability among the 4 expert annotators’ variation in judgments.

##### Win Rate

Win rate (WR) captures the proportion of evaluation instances in which one LLM’s summary is preferred over another. This metric is used to compare the relative performance of summarization LLMs.

##### Bootstrapped CIs

We perform statistical tests (bootstrapping) and report the CIs to rigorously assess the significance of our evaluation results. For each test, we generate 10,000 resamples with replacement from the original 246 data samples. The following analyses are conducted:

First, prompt EG versus baseline (95% CI for AR difference): We conduct a bootstrap resampling test (10,000 iterations) to compare the ARs between prompt-EG and the baseline prompt. Specifically, we measure the proportion of bootstrap samples where Prompt EG’s agreement with expert annotations is greater than or equal to the baseline’s. We report the 95% CI for each prompt’s AR.

Second, LLaMA 2 versus GPT-3.5 WR (95% CI for WR comparison): We similarly used bootstrap resampling to compare the WRs between LLaMA 2 and GPT-3.5. We computed the 95% CI, LLaMA 2 wins over GPT-3.5 under bootstrap resampling. This allows us to quantify the significance of LLaMA 2’s performance advantage in a nonparametric manner.

Finally, agreement between 2 annotators (95% CI for AR): This bootstrap method estimates the 95% CI for the AR between any 2 sets of labels, regardless of whether they are from experts, nonexperts, or models. Through 10,000 resamples, we measure the variability in agreement to assess the consistency between raters and the robustness of the observed alignment.

### Ethical Considerations

This study was reviewed and approved by the Queen’s University Health Sciences and Affiliated Teaching Hospitals Research Ethics Board (Tools for Research at Queen’s #6041269). The evaluation survey was administered through an approved secure online platform. An informed consent form outlining the study’s objectives, procedures, and participants’ rights was presented at the beginning of the survey. Participation was entirely voluntary, and respondents were informed of their ability to withdraw at any time before submission. All responses were collected anonymously and analyzed in aggregate to ensure privacy and confidentiality.

## Results

### Overview

To systematically assess the effectiveness of our proposed framework, we conducted a series of experiments designed to evaluate both the summarization and evaluation layers. These experiments provide a comprehensive assessment of LLMs’ summarization capabilities in medical contexts while acknowledging inherent limitations. The results are organized and presented following the validation criteria discussed earlier. Examples of GPT-4’s judgments are presented in Multimedia Appendix 2.

### LLM-Based Summarization and Evaluation

#### Comparative Performance of Llama-2-70B and Mistral-7B Across Datasets

Our first experiment focused on comparing the summarization quality of Llama-2-70B and Mistral-7B across multiple medical summarization datasets. Table 2 presents WRs against GPT-3.5 as a reference point. Llama-2-70B consistently achieved higher WRs than GPT-3.5 across all datasets, whereas Mistral-7B generally performed below GPT-3.5. These comparative results suggest Llama-2-70B’s superior performance in medical summarization tasks within our experimental framework. More detailed comparisons have been illustrated in Table 3. In Table 3, we ignored tie cases in order to provide a clearer comparative analysis. On average, Llama-2-70B achieved a WR of 65.9% against GPT-3.5 when judged by GPT-4, with a 95% CI of 48.7%-78.9%. Expert evaluations similarly favored Llama-2, with an average win rate of 68.3% and a 95% CI ranging from 50.2% to 85.2%. The expert interval lies entirely above the 50% threshold, indicating a statistically significant preference for Llama-2. While the GPT-4 interval extends above 50%, its lower bound of 48.7% is only marginally below the threshold, suggesting a favorable trend but not a statistically significant difference. Both intervals suggest that LLama-2 is more likely to outperform GPT-3.5 in real-world summarization tasks.

**Table 2.** Pairwise win rate (%) of Llama2-70b and Mistral-7b against GPT-3.5 as a reference across the different medical summarization datasets at the summarization layer of our framework. These summaries are then evaluated by GPT-4 and medical experts. The win rate indicates the percentage of instances where GPT-4 or medical experts determined that a given model produced a superior summary. The “tie” column represents cases where GPT-4 found no clear preference between the models.

Dataset and judges	GPTs’ judgment vs human experts’ judgment					
	Llama-2-70B WR^a^ (%)			Mistral-7B WR (%)		
	Llama2	GPT3.5	Tie	Mistral	GPT3.5	Tie
MEDIQA						
GPT-4	38.3	25.53	36.17	18	30	52
Expert	19.15	23.4	57.45	—^b^	—	—
MeQSum						
GPT-4	38.78	10.2	51.02	14	40	46
Expert	32.65	12.24	55.1	—	—	—
MEDIQA-ANS						
GPT-4	38	24	38	34	20	46
Expert	42	26	32	—	—	—
MEDIQA-MAS						
GPT-4	46	28	26	34	44	22
Expert	50	6	44	—	—	—
MedDialog-EN						
GPT-4	44	22	34	28	36	36
Expert	34	12	54	—	—	—
Average, mean (SD)						
GPT-4	41.02 (3.72)	21.95 (6.92)	37.04 (9.06)	25.6 (9.21)	34 (9.38)	40.4 (11.78)
Expert	35.560 (11.513)	15.928 (8.439)	48.510 (10.564)	—	—	—

a WR: win rate.

b Not available.

**Table 3.** Pairwise win rate (%) of Llama −2 against GPT-3.5 as a reference across the different medical summarization datasets at the summarization layer of our framework. These summaries are then evaluated by GPT-4 and medical experts. The win rate indicates the percentage of instances where GPT-4 or medical experts determined that LLaMA-2-70B produced a superior summary compared to GPT-3.5. We ignored tie cases in this table in order to provide a clearer comparative analysis of model preference. The 95% CIs are computed using bootstrap resampling to assess the statistical significance of the observed win rates.

Dataset and judges	LLaMA-2 WR^a^, % (95% CI)	GPT-3.5 WR, % (95% CI)
MEDIQA		
GPT-4	60.1 (42.3‐77.3)	39.9 (22.7‐57.7)
Expert	44 (22.7‐66.7)	55 (33.3‐77.3)
MeQSum		
GPT-4	79.2 (61.5‐95)	20.8 (5‐38.5)
Expert	72.7 (52.6‐90.5)	27.3 (9.5‐47.4)
MEDIQA-ANS		
GPT-4	61.3 (43.3‐78.1)	38.7 (21.9‐56.7)
Expert	61.7 (45.2‐77.8)	39.3 (22.2‐54.8)
MEDIQA-MAS		
GPT-4	62.2 (46.2‐77.4)	37.8 (22.6‐53.8)
Expert	89.3 (76‐100)	10.7 (0‐24)
MedDialog-EN		
GPT-4	66.7 (50‐82.4)	33.3 (17.6‐50)
Expert	73.9 (54.5‐90.9)	26.1 (9.1‐45.5)
Average		
GPT-4	65.9 (48.7‐78.9; SD 7.84)	34.1 (24.1‐51.3; SD 7.84)
Expert	68.32 (50.2‐85.2; SD 16.78)	31.68 (16.5‐49.8; SD 16.53)

a WR: win rate.

#### Impact of Prompt Design on Evaluation Consistency

Next, we examined how variations in prompt design affected the consistency of GPT-4’s assessments with expert judgments. Section A in Table 4 compares ARs between GPT-4 and expert majority decisions across different prompt strategies. The prompt-EG approach generally outperformed the baseline strategy, achieving higher alignment with expert consensus for most datasets. This result highlights the importance of effective prompt engineering in improving LLMs’ evaluation consistency, although the improvements vary by task type and dataset characteristics. While the bootstrapped 95% CIs for AR differences (prompt-EG−baseline) include zero in all cases, indicating that these improvements may not be statistically significant at the dataset level. However, baseline WR measures the proportion of bootstrap resamples where the baseline outperformed prompt-EG. The rate remained consistently low (eg, 7.6% on MEDIQA-QS and 14.6% on MedDialog-EN), suggesting that prompt-EG outperformed or matched the baseline in the vast majority of resampled scenarios. These findings reinforce the robustness and practical advantage of prompt-EG, even when absolute AR gains appear modest.

**Table 4.** Agreement rates between the GPT-4 judge and the majority decision of four human experts across 5 datasets, covering prompt strategies (baseline vs prompt-enhanced guidance), summarization models (GPT-3.5 and LLaMA2-70B), and position bias conditions. Section A compares agreement rates across prompts; section B reports the position bias ratio under prompt-enhanced guidance; and section C presents agreement rates for all samples and separately for samples with and without position bias. Full calculation details are provided in S1.4 in Multimedia Appendix 3.

Dataset	MEDIQA-QS	MeQSum	MEDIQA-ANS	MEDIQA-MAS	MedDialog-EN	Average, mean (SD)
Section A: prompt comparison						
Baseline AR^a^%	42.55	42.86	62.00	44.00	48.00	47.88 (8.19)
Prompt-EG^b^ AR%	51.06	46.94	57.99	48.00	54.00	51.00 (4.52)
95% CI	−4.3to 21.3	−6.1 to 14.3	−16.0 to 8.0	−6.0 to 16.0	−6.0 to 18.0	−7.7 to 15.5
Baseline WR^c^%	7.6	14.1	68.5	18.6	14.6	24.68 (24.81)
Section B: position bias						
Position bias %	12	16	36	26	34	24.8 (10.64)
Section C: AR% by position bias condition						
All samples	51.06	46.94	57.99	48.00	54.00	51.60 (4.52)
With position bias	100.00	50.00	50.00	46.15	70.59	63.35 (22.63)
Without position bias	43.90	46.34	60.52	48.65	45.45	48.97 (6.68)

a AR: agreement rate.

b EG: enhanced guidance.

c WR: win rate.

### Inaccuracy Detection and Bias Mitigation

#### Inspecting Task-Specific Trends and Position Bias

Position bias, where the order of presented summaries influences evaluation outcomes, poses a methodological challenge in LLM-based assessments. To address this, we implemented a swap-position strategy, evaluating summaries in reverse order to detect and mitigate ordering effects.

Section B in Table 4 illustrates the position bias proportion for each dataset. Interestingly, position bias was much higher in answer summarization (eg, MEDIQA-ANS and MEDIQA-MAS) and dialogue summarization (eg, MedDialog-EN) tasks compared with question summarization tasks (eg, MEDIQA-QS and MeQSum). Our analysis indicates that GPT-4 exhibits greater variability in longer and more complex summarization tasks—particularly in answer summarization and dialogue summarization. These tasks often involve layered information, implicit reasoning, and multiple clinically relevant details, making evaluation more subjective and error-prone.

Section C in Table 4 illustrates agreement patterns under different sample conditions. Our analysis revealed task-dependent patterns in performance. For *question summarization* (eg, *MEDIQA-QS*) and *dialogue summarization* (eg, *MedDialog-EN*), ARs were higher for samples with position bias. Further investigation shows that in these position-biased samples, experts themselves often produced diverse evaluations, resulting in ties or divergent decisions. This diversity suggests that summary quality in these cases was subjectively equal, with no clear superior option. GPT-4’s position bias likely stems from sampling different human perspectives from its pretraining knowledge—when summary quality is comparable, the model draws from the diverse human preferences encoded in its parameters, leading to different judgments when presentation order changes. This behavior demonstrates that GPT-4 is inconsistent as it aligns with differing human perspectives that exist within its knowledge base.

In contrast, the *answer summarization task* shows higher ARs for samples without position bias. This pattern likely stems from the more structured nature of answer summarization, which involves extracting answers directly from the provided document, thereby reducing variability in subjective interpretation. This observation is consistent with the data in Section C in Table 5, showing a higher proportion of expert consensus in answer summarization compared with the other tasks. Multimedia Appendix 4 presents illustrative examples demonstrating GPT-4’s behavior to position bias. A comprehensive analysis of the hallucination detection component within the MASA framework is presented in Multimedia Appendix 5.

**Table 5.** Human evaluation of automatic factually incorrect hallucination detection by large language models presented in percentage. The upper row in each cell reports the evaluation result, and the lower row shows the 95% CI.

Dataset	Accuracy, % (95% CI)	Precision, % (95% CI)	Sensitivity, % (95% CI)	Specificity, % (95% CI)	*F*_1_-score, % (95% CI)
Question summarization	93.0 (88.0‐97.0)	96.4 (46.5‐98.5)	65.0 (50.0, 81.2)	100 (100– 100)	71.2 (50.0‐87.7)
Dialog summarization	98.0 (94.0‐100)	99.0 (48.0‐100)	83.3 (50.0‐100)	100 (100‐100)	89.5 (49.0‐100)

In natural language generation, hallucinations refer to model-generated content that extends beyond or diverges from the information contained in the source text [15]. This broad definition often classifies even factually correct statements as hallucinations if they are not explicitly supported by the input. While this strict criterion ensures a conservative assessment of model outputs, it also conflates 2 fundamentally different cases: unsupported yet accurate domain knowledge and content that is genuinely false or misleading. To better capture this nuance, we conducted an additional evaluation focused specifically on detecting factually incorrect hallucinations (FIHs)—defined as model outputs that contradict the source text or introduce claims that conflict with established medical knowledge. Furthermore, 4 annotators collaboratively reviewed the LLM-generated summaries and resolved disagreements through discussion to determine the presence or absence of FIHs. Results are reported as macroaveraged values of FIH and non-FIH cases in terms of accuracy, precision, sensitivity, specificity, and *F*_1_-score. CIs were estimated using 10,000 bootstrap resamples based on 100 samples from a consumer health question summarization task and 50 from a dialogue summarization task.

From Table 5, our framework consistently achieved high specificity and acceptable accuracy across both tasks, indicating its strength in minimizing false alarms and preserving clinically valid information. The sensitivity scores suggest that some FIHs were missed—reflecting an area for future improvement. The CIs are wide across all metrics. This is because hallucinations were rare in both datasets—only 10 out of 100 cases in question summarization and 3 out of 50 in dialogue summarization. With such low prevalence, each correct or incorrect prediction changes accuracy, precision, and sensitivity by several percentage points, leading to greater statistical uncertainty. These variations are mainly caused by the limited sample size, not inconsistency in the detection method or framework. Overall, the results demonstrate the reliability of the proposed framework in detecting clinically relevant FIH and provide a solid basis for future work to enhance sensitivity and generalize its use in broader medical AI evaluation settings. We include more details in Multimedia Appendix 5.

### Applicability of the Framework in Real Life

#### Agreement Pattern Analysis of LLM Evaluator With Human Experts

To assess the effectiveness of LLMs as evaluators compared with humans, we computed GPT-4’s average AR with (1) each human expert across all summarization tasks, (2) at least 1 human expert across all summarization tasks for each dataset, and (3) nonexperts lacking a professional medical background and education. Then, we explored (4) AR among human evaluators and possible reasons for disagreements. Finally, we applied (5) a cross-validation strategy to ensure a fair and consistent comparison across different LLM judges.

##### Agreement of GPT-4 With Each Human Expert

Table S2 in Multimedia Appendix 3 reveals significant variations in GPT-4’s alignment with individual expert preferences when averaged over all datasets. The highest and lowest agreements of 50.54% and 36.65% with experts 3 and 4, respectively, demonstrate that LLMs inherently encapsulate diverse human perspectives from their training data and apply different viewpoints within their knowledge base when handling subjective tasks. The custom prompt-EG and models’ parameter configurations (temperature, top-p, and top-k) further influence the outcomes. This observation suggests valuable opportunities for preference customization through advanced prompt engineering.

As illustrated in Table 6, the average Cohen κ values range from 0.04 to 0.24 across datasets, with the highest agreement observed on MEDIQA-ANS (Cohen κ=0.24), which contains structured, fact-based biomedical answers. In contrast, lower Cohen κ scores, including some negative Cohen κ values in other datasets, suggest that experts often applied differing interpretive evaluation criteria or preferences, leading to variation in judgments.

**Table 6.** Pairwise Cohen κ scores between four experts across 5 medical summarization datasets.

Dataset	MEDIQA-QS	MeQSum	MEDIQA-ANS	MEDIQA-MAS	MedDialog-EN
E1					
E2	−0.23	0.20	0.40	0.32	0.10
E3	0.18	0.11	0.30	0.16	0.20
E4	0.16	0.05	0.29	−0.12	−0.07
E2					
E3	−0.15	−0.01	0.16	0.20	0.22
E4	0.16	0.36	0.14	−0.20	0.06
E3					
E4	0.15	0.14	0.16	−0.10	0.07
Average, mean (SD)	0.05 (0.18)	0.14 (0.13)	0.24 (0.10)	0.04 (0.21)	0.10 (0.11)

##### Agreement of GPT-4 With at Least 1 Human Expert

To investigate the cause of misalignment of GPT-4’s decisions against human experts and assess its potential as an evaluation tool, we measured its agreement patterns with the human experts for each dataset. Section A in Table 7 shows that GPT-4 achieves an average AR of 83.06% (95% CI 72.12%‐92.78%) with at least one of the medical experts across datasets. This finding is particularly meaningful when considered alongside the substantial disagreement observed among experts themselves. To better understand GPT-4’s evaluation limits, we conducted a detailed error analysis on 16.94% (42/246) of cases where GPT-4 did not align with any expert. We found that 4.88% (12/246) of these nonalignment cases involved instances where GPT-4 returned a tie judgment and the expert panel was evenly split (2 experts preferred Summary A, and 2 preferred Summary B). Under majority voting, such a scenario would yield a tie; however, no individual expert selected “tie,” as each expressed a definitive preference. Therefore, these cases are still considered nonalignments in this metric, as GPT-4’s tie judgment did not match any expert’s specific choice. In 9.34% (23/246) of cases, GPT-4 returned a tie while experts selected a definitive winner, often due to the summaries being extremely similar in quality, with minor differences in fluency or style. The remaining 2.8% (7/246) reflected true disagreement, where GPT-4 selected a different summary than all 4 experts, often due to overlooking nuanced clinical differences. These findings highlight the need for improved prompt strategies and clinical grounding to enhance the reliability of LLM-based evaluation.

**Table 7.** Agreement analysis among GPT-4, human experts, and nonexperts across 5 datasets in the preferred summary selection task. Section A reports the rate at which GPT-4 agrees with at least 1 expert. Section B compares agreement rates between GPT-4 and experts, GPT-4 and nonexperts, and between experts and nonexperts on simpler summarization tasks. Section C presents expert agreement rates, showing the proportion of samples where all 4 or at least 3 experts agreed. Calculation procedures are detailed in S1.2 and S1.4 in Multimedia Appendix 3.

Dataset	MEDIQA-QS	MeQSum	MEDIQA-ANS	MEDIQA-MAS	MedDialog-EN	Average rates
Section A: GPT-4 AR^a^ with ≥1 expert						
GPT-4 AR%, (95% CI)	93.62 (85.1‐100)	83.67 (73.5‐93.9)	82 (70‐92)	76 (64‐88)	80 (68‐90)	83.06 (72.12‐ 92.78; SD 5.87)
Section B: ARs among experts, nonexperts, and GPT-4						
Expert versus GPT-4 AR%, (95% CI)	51.06 (36.2‐66.0)	46.94 (32.7‐61.2)	—^b^	—	54.00 (40.0‐68.0)	50.67 (36.3‐65.1; SD 2.90)
Nonexpert versus GPT-4 AR%, (95% CI)	57.45 (42.6‐70.2)	75.51 (63.3‐87.8)	—	—	60.00 (46.0‐74.0)	64.32 (50.6‐77.3; SD 7.98)
Expert versus Nonexpert AR%, (95% CI)	42.55 (27.7‐57.4)	38.78 (26.5‐53.1)	—	—	34.00 (22.0‐48.0)	38.44 (25.4‐52.8; SD 3.50)
Section C: expert agreement with 4 and ≥3 experts						
All 4 experts agree AR%, (95% CI)	12 (4.0‐22.0)	16 (6.0‐26.0)	28 (16.0‐40.0)	18 (8.0‐30.0)	22 (12.0‐34.0)	19.2 (9.2‐30.4; SD 5.46)
≥3 experts agree AR% (95% CI)	48 (34.0‐62.0)	52 (38.0‐66.0)	68 (54.0‐80.0)	56 (42.0‐70.0)	46 (32.0‐60.0)	54.0 (40.0‐67.6; SD 7.80)

a AR: agreement rate.

b Not available.

##### Agreement of GPT-4 With Nonexperts

In addition to expert evaluations, we conducted nonexpert reviews on the shorter and simpler datasets to assess the generalizability, accessibility, and usability of the generated summaries for a broader audience. Nonexperts are identified as individuals without any professional medical background or education. While medical experts provide critical insights into accuracy and relevance, nonexperts offer a valuable perspective on clarity and comprehensibility for individuals without specialized medical knowledge. This dual evaluation approach is particularly important in health care settings, where patients and caregivers often rely on the summary of medical information. Since the nonexperts reported difficulty in identifying factual inaccuracies due to limited medical expertise for the long documents in MEDIQA-ANS and MEDIQA-MAS during the review process, we excluded these datasets in this experiment. This way, we could demonstrate the general understanding of the nonexpert population compared with the experts and GPT-4 in summarization tasks.

Section B in Table 7 reveals that GPT-4’s judgments align more closely with nonexperts (AR=64.32%, 95% CI 50.6%‐77.3%) than with experts (AR=50.67%, 95% CI 36.3%-65.1%), which is expected. This differential alignment can be attributed to GPT-4’s training methodology. During fine-tuning, GPT-4 was likely optimized using nonexpert human feedback that prioritized general comprehensibility and user satisfaction. However, GPT-4’s pretraining on diverse datasets, including medical corpora, provided it with domain knowledge that enables partial alignment with expert judgments. This explains why the AR between GPT-4 and experts (AR=50.67%, 95% CI 36.3%‐65.1%) exceeds that between experts and nonexperts (AR=38.4%, 95% CI 25.4%‐52.8%), suggesting GPT-4 occupies a middle ground between specialized and general regarding evaluation perspectives. Further analysis revealed that when GPT-4 and nonexperts favored a different summary than the experts, their preferences were often driven by surface-level detail or fluency improvements. In contrast, experts prioritized clinical fidelity over stylistic clarity. This pattern highlights the need to craft evaluation prompts that explicitly instruct LLMs to prioritize clinical accuracy alongside linguistic quality, especially in medical contexts.

##### Agreement and Disagreement Among Human Experts and Nonexperts

Section C in Table 7 highlights the variability in consensus across different expert group sizes. Notably, the average AR where all 4 experts agree with each other is only 19.2% (SD 5.46), with a 95% bootstrap CI of 9.2%‐30.4% based on 10,000 resamples. However, this rate significantly increases to 51.6% (95% CI 40%‐67.6%) when considering cases where at least 3 experts agree. This disparity underscores potential differences in expert interpretation or judgment, reflecting the complexity of achieving unanimous agreement in this task. Expert consensus was generally higher for answer summarization tasks (eg, MEDIQA-ANS and MEDIQA-MAS) than for question summarization tasks (eg, MEDIQA-QS and MeQSum), likely due to the latter’s brevity and informality, which limited content differentiation and led to more subjective and variable expert preferences.

##### Cross-Validation Strategy

**Figure 2.**
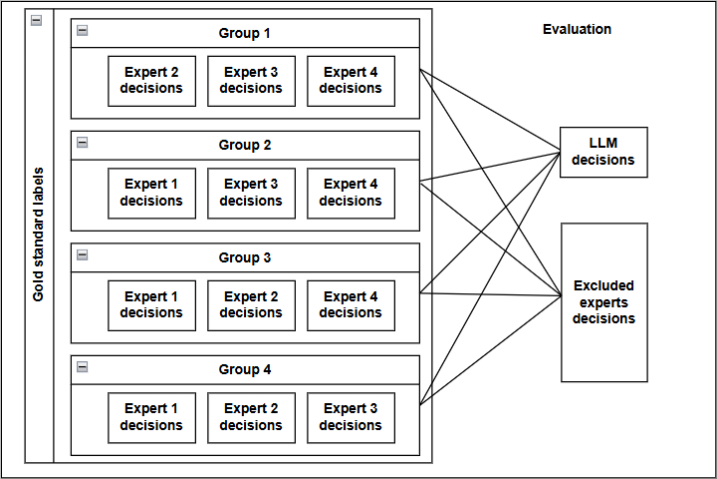
The cross-validation framework. In each iteration, 1 expert is excluded from the group of 4, and the decisions of the remaining 3 experts are used as the gold standard. GPT-4’s decisions and the excluded expert’s decisions are then compared against this gold standard, enabling a fair assessment of performance and individual alignment across all groups. LLM: large language model.

To establish a fair evaluation of ARs, we used cross-validation by systematically excluding 1 expert at a time from the group of 4, as illustrated in Figure 2. This process generated 4 groups, with the majority decision of the remaining 3 experts serving as the reference or gold standard annotation for each data point. We then compared both GPT-4’s decisions and the excluded expert’s decisions against this reference, allowing us to assess how individual human evaluators and the LLM perform relative to the same benchmark. This methodology serves multiple purposes, such as evaluating the stability of judgment distributions, identifying potential outliers within the expert group, and providing a consistent framework for comparing human and LLM performance.

Table 8 illustrates that expert 1 (excluded from group 1) achieved the highest AR of 49.87% (95% CI 36.1%‐63.3%) with other experts, while expert 3 (excluded from group 3) exhibited the lowest AR of 40.49% (95% CI 27.1%‐54.3%). The absence of extreme outliers suggests a natural distribution of expert perspectives rather than anomalous judgments. Notably, excluded experts achieved an average AR of 45.1% (SD 3.48), compared with GPT-4’s 46.95% (SD 3.44). This demonstrates that GPT-4’s evaluation capability approximates human-level consistency in this experimental context. We assessed a few newer open-source models, including Llama-3.3 and Mistral-Large, finding their performance (43.72% and 43.52%, respectively) to be slightly below GPT-4 but still within the range of human variation (lowest 40.49%). These findings suggest that current LLMs can function as supplementary evaluation tools in scenarios where expert resources are limited, although they should not be considered as replacements for human judgment.

**Table 8.** Agreement rates between GPT-4 and excluded experts in the cross-validation framework. The table shows that the excluded expert achieves a slightly higher average agreement rate (47.38%) compared with GPT-4 (46.95%), highlighting GPT-4’s close approximation to human-level performance in selecting better summaries.

Annotation group	Excluded expert, AR^a^% (95% CI)	GPT-4, AR% (95% CI)	Llama-3.3, AR% (95% CI)	Mixtral-Large, AR% (95% CI)	OpenBioLLM, AR% (95% CI)
Group 1	49.87 (36.1‐63.3)	46.02 (31.8‐59.8)	42.62 (29.2‐56.4)	48.71 (32.1‐60.5)	39.80 (26.4‐54.0)
Group 2	46.50 (30.7‐58.3)	44.33 (30.5‐58.6)	43.36 (29.6‐57.2)	44.60 (27.6‐54.8)	40.52 (26.7‐54.3)
Group 3	40.49 (27.1‐54.3)	44.63 (30.8‐58.9)	39.17 (26.6‐53.0)	38.57 (27.2‐54.4)	29.97 (18.2‐43.4)
Group 4	43.54 (30.1‐57.4)	52.81 (38.6‐66.6)	49.74 (35.9‐63.1)	42.19 (32.8‐60.8)	42.98 (29.2‐56.8)
Average	45.10 (31‐58.3; SD 3.48)	46.95 (32.9‐61.0; SD 3.44)	43.72 (30.3‐57.4; SD 3.82)	43.52 (30.0‐57.6; SD 3.69)	38.32 (25.1‐52.1; SD 4.96)

a AR: agreement rate.

Additionally, we explored OpenBioLLM-70B [12], a SOTA biomedical language model. Previous studies have reported its superior performance across a variety of biomedical NLP benchmarks, often outperforming models such as GPT-4, Gemini, Meditron-70B, Med-PaLM-1, and Med-PaLM-2. However, in our evaluation of subjective summary preference tasks, OpenBioLLM-70B achieved the lowest AR with expert judgments (38.32%), despite its domain specialization. These results suggest that while OpenBioLLM may excel in traditional biomedical NLP benchmarks, it may not be equally effective in subjective evaluation tasks, such as summary preference judgments, which require not only domain knowledge but also alignment with human evaluative heuristics like coherence and fluency. This suggests that extensive domain adaptation may come at the expense of general analytical and comparative reasoning capabilities, which are essential for tasks like pairwise summary evaluation. This trade-off highlights the importance of balancing domain expertise with general reasoning abilities when selecting or designing LLM-based evaluators.

## Discussion

### Key Observations and Implications

This study introduces a multiagent framework designed to enhance medical summarization and evaluation tasks using LLM. The first layer, the summarization layer, focuses on extracting concise and essential information from unstructured medical data, such as patient inquiries and doctor-patient conversations. This transformation enhances data clarity and prepares it for further assessment. The second layer, the evaluation layer, uses LLM judges for pairwise comparison of summaries, leveraging strategies like the *Referenced Condorcet Assessment* to reduce comparison workload and enhance accuracy. A majority-based decision-making approach, coupled with a swap-position method to mitigate position bias, ensures consistent and unbiased evaluations. This 2-layer design creates a robust framework for efficient medical summarization and evaluation, contributing to improved health care decision support.

Our findings reveal variable ARs between GPT-4 and medical experts across datasets, reflecting both the inherent subjectivity of summary evaluation and the diversity of expert perspectives. Rather than indicating a failure of the evaluation system, this variation demonstrates that qualitative assessments of summary lack a single “ground truth” even among human experts. GPT-4’s ability to align with different expert viewpoints suggests it can access multiple perspectives within its knowledge base—a capability that could be leveraged to customize and personalize evaluation systems that align with specific user preferences or institutional guidelines. Future systems can enhance robustness by incorporating multiple expert viewpoints into both the summarization and evaluation processes through prompt designs that reflect diverse reasoning patterns or ensemble-style LLM configurations that aggregate outputs from varied prompts. These techniques can reduce the impact of subjective bias and increase the reliability of automated medical NLP systems in real-world settings.

Due to the small sample size per dataset (50 samples each), we recognize that individual dataset trends should be interpreted with caution, as minor fluctuations in ARs may not be statistically meaningful. To mitigate this limitation, our study was designed to include 3 summarization tasks across 5 distinct datasets (total n=246 samples) to allow a more task-level and prompt-level comparison. Across this broader evaluation, prompt-EG consistently demonstrated improved or comparable alignment with expert judgments relative to the baseline prompt. While a slight dip was observed on the MEDIQA-ANS dataset, we consider this within the margin of expected variance given the small sample size. Importantly, the overall performance across tasks favors prompt-EG, supporting its design goal of promoting more consistent and structured evaluation across different medical summarization contexts.

Cross-validation results demonstrate that GPT-4’s ARs (45.1%) closely approximate those of individual experts when compared against the same reference standard (47.38%). This finding suggests that current LLMs can achieve human-comparable consistency in evaluation tasks, although with important limitations. In addition to GPT-4, we also evaluate several open-source models to assess their potential as transparent and controllable alternatives. While these open-source models do not surpass GPT-4 in cross-validation, the gap between their performance is relatively modest. This suggests that with task-specific tuning or further alignment, open-source evaluators may serve as viable alternatives in future clinical NLP applications where transparency and control are essential. The high alignment rate (83.06%) with at least 1 expert further indicates that GPT-4 can capture perspectives within the natural distribution of human judgment, making it potentially valuable as a complementary evaluation tool rather than a replacement for human expertise. However, in real-world clinical use, false positives (selecting hallucinated or inaccurate summaries) can mislead decision-making and compromise patient safety. False negatives (failing to identify clinically useful summaries) may result in missed insights or delayed responses. These risks underscore the need for cautious deployment, continuous human oversight, and further refinement of LLM evaluation frameworks in medical contexts.

Our findings should be interpreted in the context of medical summarization tasks—the observed alignment patterns may not generalize to other domains with different subjectivity profiles. The framework’s performance demonstrates the feasibility of LLM-assisted evaluation in specialized domains, particularly when human resources are limited or when rapid and scalable assessments are required.

The scalability and adaptability of the proposed multiagent framework make it well-suited for real-world deployment in both clinical and commercial health care environments. For instance, the summarization modules could be integrated into electronic health record systems to help clinicians rapidly review patient histories and incoming referrals. In the context of insurance and billing, the framework could enhance audit workflows by condensing extensive clinical documentation for claim validation. Furthermore, the LLM-based evaluation layer could act as a quality control module, which can identify the summaries that lack clarity or medical consistency, coherence, fluency, and relevance. Textbox 1 presents a summary of the key findings and recommendations.

Textbox 1. **Summary of key findings and recommendations.**

**Key findings**

- GPT-4 demonstrates alignment levels comparable with individual medical experts and captures a range of expert viewpoints.
- Prompt-enhanced guidance improves evaluation consistency compared with the baseline prompts, especially in structured summarization tasks.
- Even experts’ discussion cannot align with each other.

**Recommendations**

- Large language models comparable with GPT-4 can complement expert reviewers in clinical or admin workflows.
- GPT-4 can support doctors by streamlining summary review and reducing workload.

### Limitations

Despite the promising results, several limitations need to be considered.

First, our study involved a limited number of medical experts, which may not capture the full diversity of professional opinions in medical summarization tasks. We aimed to recruit as many experts as possible, based on the understanding that a larger and more diverse panel could help establish more robust human agreement benchmarks. However, we encountered significant challenges during recruitment. These included scheduling conflicts, limited availability, and the high cognitive effort required for detailed evaluation. As a result, only 4 experts participated in this study. This limitation highlights the need for scalable expert engagement strategies in future work.

- We evaluated a limited set of LLMs. As model architectures and training methodologies evolve rapidly, expanding the range of assessed models would provide more comprehensive insights into the capabilities and limitations of different approaches.
- While our framework shows promise for evaluation tasks, the quality of generated summaries remains variable. Enhancing summary quality through domain-specific fine-tuning, retrieval-augmented generation (RAG), hybrid human-AI workflows, or leveraging our evaluation system as a reward signal for reinforcement learning approaches to automatically improve the quality of summaries presents important directions for future research.

Second, we evaluated a limited set of LLMs. As model architectures and training methodologies evolve rapidly, expanding the range of assessed models would provide more comprehensive insights into the capabilities and limitations of different approaches.

Third, while our framework shows promise for evaluation tasks, the quality of generated summaries remains variable. Enhancing summary quality through domain-specific fine-tuning, retrieval-augmented generation, hybrid human-AI workflows, or leveraging our evaluation system as a reward signal for reinforcement learning approaches to automatically improve the quality of summaries presents important directions for future research.

Fourth, although prompt-EG was designed to output confidence levels (ie, low, medium, and high) alongside its pairwise judgments, these confidence scores were not analyzed in this study. We acknowledge that a confidence estimate could serve as a valuable signal in future evaluation pipelines. For example, confidence scores might be used to weight the strength of individual judgments, which can resolve conflicts among multiple evaluators. Incorporating and systematically analyzing these confidence levels present a promising direction for future research.

Fifth, while the evaluation metrics were computed over a randomly selected set of 50 instances per dataset, these results are intended to illustrate relative performance patterns rather than make definitive statistical generalizations. Future work could expand the evaluation set and incorporate confidence intervals or statistical testing to strengthen robustness claims.

Sixth, our evaluation metrics focus primarily on ARs rather than more nuanced measures for evaluating various qualitative aspects of summaries. Developing multidimensional evaluation frameworks that assess factual accuracy, coherence, relevance, and accessibility separately could provide more granular insights into model performance.

Finally, this study is limited to English-language datasets primarily originating from US-based clinical and consumer health sources. Therefore, the generalizability of the proposed framework to other languages and health care systems remains an open question.

### Conclusions

This study presents a multiagent framework, MASA, leveraging LLMs for medical summarization and evaluation. By integrating a summarization layer and an evaluation layer, our system addresses key challenges in processing unstructured medical data and ensuring accuracy, coherence, and relevance in generated summaries. The introduction of the *Referenced Condorcet Assessment*, swap position approach, and enhanced prompt designs like prompt-EG demonstrates how methodological refinements can mitigate common issues such as position bias.

Our findings demonstrate that current LLMs can achieve evaluation consistency comparable with individual human experts, although with important limitations and context dependencies. This suggests the feasibility of LLM-assisted evaluation in specialized domains, particularly when human resources are limited or when rapid, scalable assessment is required.

Future work should focus on expanding expert panels, evaluating diverse LLM architectures, enhancing summary quality through domain-specific techniques, and developing more nuanced evaluation metrics. By addressing these challenges, we can move toward more robust, transparent, and effective AI systems for medical information processing—systems that complement rather than replace human expertise while maintaining alignment with evolving capabilities in language model technology.

## Supplementary material

10.2196/75932 Multimedia Appendix 1 Prompt design for the summarization layer and evaluation layer.

10.2196/75932 Multimedia Appendix 2 Examples of data samples for each task, large language model summaries, and GPT-4’s judgments.

10.2196/75932 Multimedia Appendix 3 Detailed algorithms for the calculations.

10.2196/75932 Multimedia Appendix 4 Examples that demonstrate GPT-4’s behavior in summary evaluation tasks, including position bias and hallucination detection.

10.2196/75932 Multimedia Appendix 5 Detailed analysis of the hallucination detection component of multiagent summarization and auto-evaluation framework.

## Abbreviations

AI: artificial intelligence
AR: agreement rate
EG: enhanced guidance
FIH: factually incorrect hallucination
LLM: large language model
MASA: multiagent summarization and auto-evaluation
NLP: natural language processing
RCA: Referenced Condorcet Assessment
SOTA: state-of-the-art
WR: win rate
