# Supplementary material for: A Multiagent Summarization and Auto-Evaluation Framework for Medical Text: Development and Evaluation Study
Source: JMIR AI. 2025 Dec 16;4:e75932. doi: 10.2196/75932 (PMC12707800; doi:10.2196/75932)
Supplement: Multimedia Appendix 1 [file ai-v4-e75932-s001.docx]

# Appendix 1: Prompt and LLMs’ Parameter Design

This document demonstrates the prompt design for the summarization and evaluation layers for the 3 tasks on consumer health question summarization, biomedical answer summarization and patient-doctor dialog summarization. We use the same prompt for the summarization layer for all LLMs to perform a fair evaluation of the generated summaries. In the evaluation layer, we use both the baseline prompt and our proposed prompt with enhanced guidance (Prompt-EG) to evaluate the generated summaries using GPT-4 as the judge.

**Table A1.1. Prompt design for the summarization layer**.

| **Dataset** | **Prompt for the Summarization Layer** |
| --- | --- |
| Consumer Health Question Summarization | Rewrite the following question in a short and concise form: [QUESTION] |
| Biomedical Answer Summarization | Write a very short and concise summary of the following article based on the question given below: [Document] |
| Dialog Summarization | Write a very short and concise one line summary of the following dialogue as an informal question in a healthcare forum: [DIALOGUE] |

**Table A1.2. Prompt design for the evaluation layer: Prompt-baseline.**

| **Dataset** | **Prompt-baseline for the Evaluation Layer** |
| --- | --- |
| Consumer Health Question Summarization | Please act as an impartial judge and evaluate the quality of the summarized question provided by two AI assistants to the user question displayed below. You should choose the assistant that follows the user’s instructions and give a better summarized question. Your evaluation should consider factors such as the relevance, coherence, fluency, consistency. Begin your evaluation by comparing the two summarized question and provide a  short explanation. Avoid any position biases and ensure that the order in which the summarizations were presented does not influence your decision. Do not allow the length of the summarized question to influence your evaluation. Do not favor certain names of the assistants. Be as objective as possible. After providing your explanation, output your final verdict by strictly following this format: "[[A]]" if assistant A is better, "[[B]]" if assistant B is better, and "[[C]]" for a tie |
| Biomedical Answer Summarization | Please act as an impartial judge and evaluate the quality of the summarized answer provided by two AI assistants based on the provided referenced document to the user question displayed below. You should choose the assistant that follows the user’s instructions and give a better summarized answer. Your evaluation should consider factors such as the relevance, coherence, fluency, consistency. Begin your evaluation by comparing the two summarized answer and provide a short explanation. Avoid any position biases and ensure that the order in which the summarizations were presented does not influence your decision. Do not allow the length of the summarized answer to influence your evaluation. Do not favor certain names of the assistants. Be as objective as possible. After providing your explanation, output your final verdict by strictly following this format: "[[A]]" if assistant A is better, "[[B]]" if assistant B is better, and "[[C]]" for a tie" |
| Dialog Summarization | Please act as an impartial judge and evaluate the quality of summarized patients' queries generated by two AI assistants in response to a given patient and doctor conversation from a healthcare forum. Two AI assistants should only focus on patients' query and ignore doctors’ responses. Your evaluation should consider factors such as the relevance, coherence, fluency, consistency. Begin your evaluation by comparing the two summarized question and provide a short explanation. Avoid any position biases and ensure that the order in which the summarizations were presented does not influence your decision. Do not allow the length of the summarized question to influence your evaluation. Do not favor certain names of the assistants. Be as objective as possible. After providing your explanation, output your final verdict by strictly following this format: "[[A]]" if assistant A is better, "[[B]]" if assistant B is better, and "[[C]]" for a tie |

**Table A1.3. Prompt design for the evaluation layer: Prompt-EG.**

| **Dataset** | **Prompt-EG for the Evaluation Layer** |
| --- | --- |
| Consumer Health Question Summarization | In this task, you are required to adopt the role of a medical professional, and act as an unbiased evaluator of the quality of summarized responses generated by two AI assistants in response to a given user question from the medical domain. Your evaluation should be grounded in four critical aspects: relevance, coherence, fluency, and consistency. Please approach the evaluation with the following guidelines:  **Relevance:** Selection of important content from the source. The summary should include only important information from the source document. Annotators were instructed to penalize summaries which contained redundancies and excess information.  **Coherence:** The collective quality of all sentences. We align this dimension with the DUC quality question of structure and coherence whereby "the summary should be well-structured and well-organized. The summary should not just be a heap of related information, but should build from sentence to a coherent body of information about a topic."  **Fluency:** Selection of important content from the source. The summary should include only important information from the source document. Annotators were instructed to penalize summaries which contained redundancies and excess information.  **Consistency:** The factual alignment between the summary and the summarized source. A factually consistent summary contains only statements that are entailed by the source document. Annotators were also asked to penalize summaries that contained hallucinated facts.  **Avoid Positional Bias:** Start your evaluation without considering the order in which the summaries are presented. Assess each summary on its own merits before comparing them directly.  **Compression and Detail:** Evaluate the quality of the summarization not just based on the amount of detail it provides, but also on how effectively it compresses the essential information from the original inquiry. A high-quality summary should balance detail and brevity, adhering to the user's request for summarization without unnecessary elaboration.  **Detection of Hallucinations:** Pay particular attention to the accuracy of the content within each summary. Assess whether the summary introduces information not present in the original question ('hallucinations') and factor this into your evaluation. The ability to remain faithful to the provided information is paramount.  **Objective Evaluation:** Disregard the names of the AI assistants to ensure an unbiased assessment. Your evaluation must focus solely on the content and quality of the summaries.  Please identify the parts in the original long question that might cause confusion during summarization and pay more attention on factual consistency between source question and summaries.  After reviewing the summaries, compare them based on the aforementioned criteria. Provide a brief explanation for your choice, focusing on how each summary aligns with the user's instructions and the evaluation criteria. Finally, conclude with your final verdict using the specified format: "[[A]]" if assistant A's summary is superior, "[[B]]" if assistant B's summary is superior, and "[[C]]" for a tie. Your explanation should justify your decision by referencing specific elements of relevance, coherence, fluency, and consistency in the summaries."  In addition, you are also asked to rate the confidence level of your conclusion. Your confidence level should reflect how certain you are about the decision made, based on the evaluation criteria of relevance, coherence, fluency, and consistency. Consider the following scale for your confidence rating:  **Low Confidence:** The decision was difficult to make, with both summaries presenting significant strengths and weaknesses that are closely matched. There might be substantial ambiguity or a lack of clear differentiation in the quality of the summaries according to the evaluation criteria.  **Medium Confidence:** The decision is supported by noticeable differences between the summaries in one or more evaluation criteria, but there are still some uncertainties or close calls in the assessment that prevent a high confidence rating.  **High Confidence:** The decision is made with a clear and definitive assessment, where one summary distinctly outperforms the other across most or all of the evaluation criteria, leading to minimal doubt about the conclusion.  Please state your confidence level in this conclusion by using the specified format: "[[Low]]" if the confidence level is low, "[[Medium]]" if the confidence level is medium, and "[[High]]" if the confidence level is high. |
| Biomedical Answer Summarization | In this task, you will adopt the role of a medical professional and act as an unbiased evaluator to determine which of two summarized responses better addresses a given user question in the medical domain. You must consider the provided reference document to ensure the responses align accurately with the context and information it contains. Base your evaluation on four critical criteria: relevance, coherence, fluency, and consistency.  **Relevance:** Selection of important content from the source. The summary should include only important information from the source document. Annotators were instructed to penalize summaries which contained redundancies and excess information.  **Coherence:** The collective quality of all sentences. We align this dimension with the DUC quality question of structure and coherence whereby "the summary should be well-structured and well-organized. The summary should not just be a heap of related information, but should build from sentence to a coherent body of information about a topic."  **Fluency:** Selection of important content from the source. The summary should include only important information from the source document. Annotators were instructed to penalize summaries which contained redundancies and excess information.  **Consistency:** The factual alignment between the summary and the summarized source. A factually consistent summary contains only statements that are entailed by the source document. Annotators were also asked to penalize summaries that contained hallucinated facts.  **Avoid Positional Bias:** Start your evaluation without considering the order in which the summaries are presented. Assess each summary on its own merits before comparing them directly.  **Compression and Detail:** Evaluate the quality of the summarization not just based on the amount of detail it provides, but also on how effectively it compresses the essential information from the original inquiry. A high-quality summary should balance detail and brevity, adhering to the user's request for summarization without unnecessary elaboration.  **Detection of Hallucinations:** Pay particular attention to the accuracy of the content within each summary. Assess whether the summary introduces information not present in the referenced document ('hallucinations') and factor this into your evaluation. The ability to remain faithful to the provided information is paramount.  **Objective Evaluation:** Disregard the names of the AI assistants to ensure an unbiased assessment. Your evaluation must focus solely on the content and quality of the summaries.  Please identify the parts in the question and referenced document that might cause confusion during summarization and pay more attention on factual consistency between source question, referenced document and summaries.  After reviewing the summaries, compare them based on the aforementioned criteria. Provide a brief explanation for your choice, focusing on how each summary aligns with the user's instructions and the evaluation criteria. Finally, conclude with your final verdict using the specified format: "[[A]]" if assistant A's summary is superior, "[[B]]" if assistant B's summary is superior, and "[[C]]" for a tie. Your explanation should justify your decision by referencing specific elements of relevance, coherence, fluency, and consistency in the summaries."  In addition, you are also asked to rate the confidence level of your conclusion. Your confidence level should reflect how certain you are about the decision made, based on the evaluation criteria of relevance, coherence, fluency, and consistency. Consider the following scale for your confidence rating:  **Low Confidence:** The decision was difficult to make, with both summaries presenting significant strengths and weaknesses that are closely matched. There might be substantial ambiguity or a lack of clear differentiation in the quality of the summaries according to the evaluation criteria.  **Medium Confidence:** The decision is supported by noticeable differences between the summaries in one or more evaluation criteria, but there are still some uncertainties or close calls in the assessment that prevent a high confidence rating.  **High Confidence:** The decision is made with a clear and definitive assessment, where one summary distinctly outperforms the other across most or all of the evaluation criteria, leading to minimal doubt about the conclusion.  Please state your confidence level in this conclusion by using the specified format: "[[Low]]" if the confidence level is low, "[[Medium]]" if the confidence level is medium, and "[[High]]" if the confidence level is high. |
| Dialog Summarization | In this task, you are required to adopt the role of a medical professional, and act as an unbiased evaluator of the quality of summarized patients' queries generated by two AI assistants in response to a given patient and doctor conversation from a healthcare forum. Two AI assistants should only focus on patients' query, and ignore doctors' responses. Your evaluation should be grounded in four critical aspects: relevance, coherence, fluency, and consistency. Please approach the evaluation with the following guidelines:  **Relevance:** Selection of important content from the source. The summary should include only important information from the source document. Annotators were instructed to penalize summaries which contained redundancies and excess information.  **Coherence:** The collective quality of all sentences. We align this dimension with the DUC quality question of structure and coherence whereby "the summary should be well-structured and well-organized. The summary should not just be a heap of related information, but should build from sentence to a coherent body of information about a topic."  **Fluency:** Selection of important content from the source. The summary should include only important information from the source document. Annotators were instructed to penalize summaries which contained redundancies and excess information.  **Consistency:** The factual alignment between the summary and the summarized source. A factually consistent summary contains only statements that are entailed by the source document. Annotators were also asked to penalize summaries that contained hallucinated facts.  **Avoid Positional Bias:** Start your evaluation without considering the order in which the summaries are presented. Assess each summary on its own merits before comparing them directly.  **Compression and Detail:** Evaluate the quality of the summarization not just based on the amount of detail it provides, but also on how effectively it compresses the essential information from the original inquiry. A high-quality summary should balance detail and brevity, adhering to the user's request for summarization without unnecessary elaboration.  **Detection of Hallucinations:** Pay particular attention to the accuracy of the content within each summary. Assess whether the summary introduces information not present in the original question ('hallucinations') and factor this into your evaluation. The ability to remain faithful to the provided information is paramount.  **Objective Evaluation:** Disregard the names of the AI assistants to ensure an unbiased assessment. Your evaluation must focus solely on the content and quality of the summaries.  Please identify the parts in the original long question that might cause confusion during summarization and pay more attention on factual consistency between source question and summaries.  After reviewing the summaries, compare them based on the aforementioned criteria. Provide a brief explanation for your choice, focusing on how each summary aligns with the user's instructions and the evaluation criteria. Finally, conclude with your final verdict using the specified format: "[[A]]" if assistant A's summary is superior, "[[B]]" if assistant B's summary is superior, and "[[C]]" for a tie. Your explanation should justify your decision by referencing specific elements of relevance, coherence, fluency, and consistency in the summaries."  In addition, you are also asked to rate the confidence level of your conclusion. Your confidence level should reflect how certain you are about the decision made, based on the evaluation criteria of relevance, coherence, fluency, and consistency. Consider the following scale for your confidence rating:  **Low Confidence:** The decision was difficult to make, with both summaries presenting significant strengths and weaknesses that are closely matched. There might be substantial ambiguity or a lack of clear differentiation in the quality of the summaries according to the evaluation criteria.  **Medium Confidence:** The decision is supported by noticeable differences between the summaries in one or more evaluation criteria, but there are still some uncertainties or close calls in the assessment that prevent a high confidence rating.  **High Confidence:** The decision is made with a clear and definitive assessment, where one summary distinctly outperforms the other across most or all of the evaluation criteria, leading to minimal doubt about the conclusion.  Please state your confidence level in this conclusion by using the specified format: "[[Low]]" if the confidence level is low, "[[Medium]]" if the confidence level is medium, and "[[High]]" if the confidence level is high. |
