# Supplementary material for: A Multiagent Summarization and Auto-Evaluation Framework for Medical Text: Development and Evaluation Study
Source: JMIR AI. 2025 Dec 16;4:e75932. doi: 10.2196/75932 (PMC12707800; doi:10.2196/75932)
Supplement: Multimedia Appendix 2 [file ai-v4-e75932-s002.docx]

# Appendix 2: Judgement of Summaries

This document demonstrates the experiment on using two different LLMs (Assistant A and Assistant B) to generate summaries of (Task 1) health questions, (Task 2) answers from biomedical data, and (Task 3) patient-doctor dialogues with GPT-4 acting as a judge for selecting the better summary. Examples from Tasks 1, 2, and 3 are presented below

# Task 1:

Consumer Health Question Summarization

## Question for summarization:

I’m 30 years old and when I was 23 my period wasnt coming every month, and I still don’t get my period, last time I got it was 4 months ago and I’m really worried.

## Assistant A’s summary:

Why am I experiencing irregular menstrual cycles and a lack of periods at 30 years old?

## Assistant B’s summary:

Concise version:\n\nI'm 30, haven't had a period in 7 years, and am worried. What's wrong?

## GPT-4’s Judgement (Prompt-EG):

**Relevance:**

- Assistant A's summary captures the essence of the user's concern by directly addressing the issue of irregular menstrual cycles and the current lack of periods at the age of 30. It accurately reflects the user's situation without adding or omitting significant details.

- Assistant B's summary, while concise, slightly misinterprets the user's original statement. The user did not indicate a complete absence of periods for 7 years but mentioned that the last period was 4 months ago and that periods have been irregular since the age of 23. This introduces a minor factual inconsistency.

**Coherence:**

- Assistant A's summary is coherent, presenting the user's concern in a clear, question format that logically follows from the information provided.

- Assistant B's summary is also coherent but less accurate due to the misunderstanding of the user's menstrual history.

**Fluency:**

- Both summaries are fluent, with Assistant A's summary being structured as a clear question and Assistant B's as a concise statement. There are no grammatical or syntactical errors in either summary.

**Consistency:**

- Assistant A's summary is consistent with the original question, accurately reflecting the user's experience with irregular menstrual cycles and the recent absence of periods.

- Assistant B's summary, while generally consistent with the theme of concern over menstrual irregularities, introduces a slight inconsistency by suggesting a continuous 7-year absence of periods, which is not supported by the original question.

**Compression and Detail:**

- Assistant A effectively compresses the essential information into a question format, maintaining the detail necessary to understand the user's concern.

- Assistant B's attempt at compression results in a loss of important detail regarding the timing and nature of the menstrual irregularities.

**Detection of Hallucinations:**

- There are no hallucinations in Assistant A's summary.

- Assistant B's summary contains a minor hallucination regarding the duration of the period absence.

**Objective Evaluation:**

Based on the evaluation criteria, Assistant A's summary is superior due to its accuracy, coherence, and consistency with the original question. Assistant B's summary, while concise, introduces a factual error that could lead to misunderstanding the user's medical history.

**Conclusion:** [[A]]

**Confidence Level:** [[High]]

## GPT-4’s Judgement (Prompt-baseline):

Assistant A's summarized question captures the essence of the user's concern by specifically mentioning the ongoing issue of irregular menstrual cycles and the current age of the user, which provides context and clarity. It accurately reflects the user's situation of experiencing irregular periods since the age of 23 and the continuation of this issue up to the present age of 30. This summary is coherent, relevant, and directly addresses the user's request for understanding the cause behind their menstrual irregularities.

Assistant B's summarized question, while concise, slightly misinterprets the user's original statement. It suggests that the user hasn't had a period in 7 years, which is not explicitly stated in the user's question. The user mentioned that the last period was 4 months ago and that the irregularity started at 23, not that periods have been completely absent for 7 years. This summary, although brief and to the point, lacks the accuracy and detail provided by Assistant A in capturing the user's situation.

Based on these observations, Assistant A's summary is more accurate, relevant, and faithful to the user's original question. It maintains the context and specifics of the user's concern, making it the better summary.

**Final Verdict:** [[A]]

# Task 2:

Biomedical Answer Summarization

## Document:

Retinitis pigmentosa (RP) is a group of rare, genetic disorders that involve a breakdown and loss of cells in the retina‚Äî which is the light sensitive tissue that lines the back of the eye. Common symptoms include difficulty seeing at night and a loss of side (peripheral)¬†vision. The retina is the light-sensitive tissue at the back of the eye that contains photoreceptors and other cell¬†types\n, Available treatments aim to slow the progression of the disease and primarily include light avoidance and the use of low-vision aids. Some practitioners also consider vitamin A as a possible treatment option. However, taking too much vitamin A can be toxic and the effects of vitamin A on the disease appear to be relatively weak.[4] Studies have explored potential treatment with DHA, an omega-3 fatty acid naturally found in fish. While DHA is known to play a structural role in retinal cells, more research is needed to determine whether supplements should be recommended.[2] Current research is focused on the development of new treatments including gene therapy, retinal transplantation, and the use of a retinal prosthesis. Stem cell transplantation would involve the injection and integration of stem cells into the retina, in hopes these cells will replace dead cells and provide the missing enzymes and chemicals needed for sight. Gene therapy could potentially be used when the disease-causing mutation is known and would aim to restore production of the missing or abnormal protein. Studies with retinal prosthetics have tested devices that transform light into electrical signals that can be sent directly to the inner retina and brain, avoiding the diseased part of the outer retina. Though challenges remain, preliminary research into these technologies has been promising.[2][3] FDA-Approved Treatments The medication(s) listed below have been approved by the Food and Drug Administration (FDA) as orphan products for treatment of this condition. Learn more orphan products. National Library of Medicine Drug Information Portal\n, Retinitis pigmentosa (RP) is a group of inherited eye diseases that affect the light-sensitive part of the eye (retina). RP causes cells in the retina to die, causing progressive vision loss. The first sign of RP usually is night blindness. As the condition progresses, affected individuals develop tunnel vision (loss of peripheral vision), and eventually loss of central vision. RP may be caused by mutations in any of at least 50 genes. Inheritance can be autosomal dominant, autosomal recessive, or X-linked .[1] Treatment options to slow the progression of vision loss include light avoidance, use of low-vision aids, and vitamin A supplementation. Researchers are working to develop new treatment options for the future such as gene therapy, stem cell transplantation and prosthetic implants.[2][3]\n, Vitamin A palmitate Omega-3 fatty acid supplements Lutein plus zeaxanthin supplements Sometimes, drugs called carbonic anhydrase inhibitors Sometimes, surgically implanted computer chips No treatment can reverse the damage caused by retinitis pigmentosa. Vitamin A palmitate may help slow the progression of the disorder in some people. Supplements with an omega-3 fatty acid (for example, docosahexaenoic acid) and a combination of lutein plus zeaxanthin may also slow the rate of vision loss. People who have swelling of the retina (macular edema), drugs called carbonic anhydrase inhibitors (usually acetazolamide or dorzolamide) are given by mouth or as eye drops. A new gene-therapy drug called voretigene neparovovec-ryzl may restore some vision to people with a particular mutation, provided they still have some retinal cells capable of surviving and reproducing. People who have lost all vision or almost all vision can be given a surgically implanted computer chip in the eye, which can restore some perception of light and shapes.\n

## Question:

Is there any help for retinitis pigmentosa?

## Assistant A’s summary:

Current treatment options for retinitis pigmentosa (RP) aim to slow the progression of the disease and include light avoidance, low-vision aids, and possibly vitamin A supplementation. Research is also focused on new treatments such as gene therapy, stem cell transplantation, and retinal prosthetics. While there are no options to reverse the damage caused by RP, there are some promising developments in treatment, such as gene therapy and prosthetic implants.

## Assistant B’s summary:

There are limited treatments available for retinitis pigmentosa (RP), a group of rare, genetic disorders that affect the retina and cause progressive vision loss. Current treatments aim to slow the progression of the disease and include light avoidance, low-vision aids, and vitamin A supplementation. Researchers are exploring new treatment options, such as gene therapy, stem cell transplantation, and retinal prosthetics, but these are still in the experimental stages. While vitamin A supplements may help slow the progression of the disorder in some people, there is no treatment that can reverse the damage caused by RP.

## GPT-4’s Judgement (Prompt-EG):

Both summaries provide a concise overview of the available treatments for retinitis pigmentosa (RP) and mention ongoing research into potential future treatments. However, there are differences in how they present this information, affecting their relevance, coherence, fluency, and consistency with the referenced document.

****Relevance:****

- Both summaries effectively highlight the main treatments and research directions for RP, including light avoidance, low-vision aids, vitamin A supplementation, gene therapy, stem cell transplantation, and retinal prosthetics.

- Assistant B slightly edges out in relevance by explicitly stating that the treatments aim to slow the progression of the disease and that no treatment can reverse the damage, which is a crucial point made in the referenced document.

****Coherence:****

- Assistant A's summary is coherent, presenting the information in a logical order that is easy to follow.

- Assistant B's summary is also coherent and benefits from a slightly clearer explanation of the current state of treatments and the experimental nature of new treatments, enhancing its coherence.

****Fluency:****

- Both summaries are fluent, with no grammatical or syntactical errors. They are well-written and easy to understand.

****Consistency:****

- Assistant A's summary is consistent with the referenced document but lacks specific details about the experimental nature of new treatments and the FDA-approved treatments.

- Assistant B's summary is more consistent with the referenced document, mentioning the experimental stages of new treatments and the lack of treatments to reverse RP damage. However, it omits details about specific supplements and FDA-approved treatments mentioned in the document.

****Detection of Hallucinations:****

- Neither summary introduces hallucinated facts; all mentioned treatments and research directions are supported by the referenced document.

****Conclusion:****

Based on the evaluation criteria, **[[B]]** Assistant B's summary is superior because it slightly better captures the essence of the referenced document, especially in terms of relevance and consistency, by explicitly stating the limitations of current treatments and the experimental nature of potential future treatments. Assistant B's summary also maintains high coherence and fluency, similar to Assistant A's summary, but with a slight edge in presenting the information in a way that might be more useful to someone seeking an understanding of RP treatments.

****Confidence Level:** [[Medium]]** - The decision is supported by noticeable differences in relevance and consistency, though both summaries are closely matched in fluency and coherence.

## GPT-4’s Judgement (Prompt-baseline):

Both Assistant A and Assistant B provide summaries that accurately reflect the content of the referenced document regarding treatments for retinitis pigmentosa (RP). Both summaries mention the current treatment options aimed at slowing the disease's progression, including light avoidance, low-vision aids, and vitamin A supplementation. They also highlight ongoing research into potential future treatments such as gene therapy, stem cell transplantation, and retinal prosthetics.

Assistant A's summary is concise and mentions the lack of options to reverse RP damage but highlights promising developments in gene therapy and prosthetic implants. Assistant B's summary is slightly more detailed, explicitly stating that the treatments available are limited and that the new treatment options are still in experimental stages. It also specifically notes that vitamin A supplements may help slow the disorder's progression in some people, but no treatment can reverse RP's damage.

The main difference lies in Assistant B's explicit mention that the new treatments are in experimental stages and the clarification about the role of vitamin A supplements, which adds a bit more depth to the understanding of the current state of RP treatments. This detail might be particularly valuable for someone seeking a comprehensive overview of the treatment landscape for RP.

Given the criteria of relevance, coherence, fluency, and consistency, both summaries are well-crafted. However, Assistant B's inclusion of the experimental status of new treatments and the specific note about vitamin A supplements provides a slightly more comprehensive understanding of the current and potential future treatment landscape for RP.

**Final Verdict:** [[B]]

# Task 3:

Patient-Doctor Dialog Summarization

## Dialog for summarization:

Patient:

Hi doctor,I have been suffering from preliminary chronic urticaria for unknown causes for the last 13 years. It is not responding to antihistamine treatment for the past six years. Sometimes, it gets as severe as I have to use Corticosterone injection (Diprofos). All blood analysis showed no cause. Dermatologist started to recommend immunity treatment such as Arpimune, which is better but very slow. Finally an immunity and allergy doctor recommended Xolair (Omalizumab) for six months. Shall I use it?

Doctor:

Hi. I understand your concern. Chronic idiopathic urticaria is a type of urticaria (wheals). It is a disturbing illness and difficult to manage. Most urticaria patients benefit from prolonged antihistamines and some may require immune suppressing drugs like Cyclosporine to control urticaria, which were also given to you. When the relief is not satisfactory from all these options, the physicians opt for a biologic medicine called Omalizumab. This medicine prevents the release of urticaria causing molecules in your body. It is considered safe and has proven efficacy in appropriate subjects. It will be given under a physician's supervision and side effects will be monitored. It has helped many patients to attain a longer symptom free periods. You can go ahead comfortably with the treatment. For further doubts consult a dermatologist online --> <https://www.icliniq.com/ask-a-doctor-online/dermatologist>

## Assistant A’s summary:

Should I consider using Xolair (Omalizumab) for my chronic urticaria that has not responded to antihistamines and other treatments?

## Assistant B’s summary:

Should I use Xolair (Omalizumab) for my chronic idiopathic urticaria that has not responded to other treatments?

## GPT-4’s Judgement (Prompt-EG):

**Relevance:**

Both summaries are highly relevant to the original query, focusing on the patient's question about the use of Xolair (Omalizumab) for treating chronic urticaria that has not responded to other treatments. Assistant A includes the detail that antihistamines and other treatments have been ineffective, which is a crucial part of the patient's history. Assistant B mentions "chronic idiopathic urticaria," which aligns closely with the doctor's diagnosis, adding specificity to the type of urticaria being discussed.

**Coherence:**

Assistant A's summary is coherent, presenting the patient's situation and question in a straightforward manner. Assistant B's summary is also coherent and benefits from the use of the term "chronic idiopathic urticaria," which provides a clear and medically accurate context for the question.

**Fluency:**

Both summaries are fluent, with no grammatical or syntactical issues. They are clear and easy to understand, presenting the patient's query in a concise manner.

**Consistency:**

Assistant A's summary is consistent with the original query, accurately reflecting the patient's history of treatment resistance and the consideration of Xolair (Omalizumab). Assistant B's summary is also consistent but adds the term "chronic idiopathic urticaria," which is a detail that aligns well with the doctor's response and the nature of the patient's condition, enhancing the factual accuracy of the summary.

**Compression and Detail:**

Assistant A provides a good balance of detail and compression, mentioning the lack of response to antihistamines and other treatments. Assistant B, while slightly less detailed about the previous treatments, includes the term "chronic idiopathic urticaria," which adds valuable specificity to the summary.

**Detection of Hallucinations:**

Neither summary introduces hallucinated facts; both stick closely to the information provided in the original conversation.

**Objective Evaluation:**

Both summaries are of high quality, but Assistant B's inclusion of "chronic idiopathic urticaria" provides a slight edge in terms of medical specificity and relevance to the patient's condition. This term directly references the nature of the urticaria being discussed, which is a detail that enhances the summary's relevance and accuracy.

**Final Verdict:**

[[B]] - Assistant B's summary is superior due to its inclusion of the specific type of urticaria, which adds relevance and specificity to the patient's query.

**Confidence Level:**

[[Medium]] - The decision is supported by the slight difference in the inclusion of specific medical terminology by Assistant B, which enhances the summary's relevance and accuracy. However, both summaries are closely matched in quality, leading to a medium confidence rating.

## GPT-4’s Judgement (Prompt-baseline):

Both Assistant A and Assistant B have provided a concise and relevant summary of the patient's query. The main difference between the two summaries lies in the specificity of the condition and treatments mentioned:

- Assistant A specifies the condition as "chronic urticaria" and notes that it has not responded to "antihistamines and other treatments." It also mentions the patient's history of not responding to antihistamine treatment explicitly.

- Assistant B uses the term "chronic idiopathic urticaria," which is more specific and accurate, reflecting the doctor's diagnosis. It also mentions that the condition has not responded to "other treatments" without specifying what those treatments are, which is less detailed compared to Assistant A's mention of antihistamines explicitly.

Both summaries are coherent, fluent, and consistent with the patient's original query. The choice between them hinges on the value placed on the specificity of the condition (idiopathic vs. general chronic urticaria) and the explicit mention of antihistamines as part of the treatment history.

Given the importance of accurately describing the medical condition and the treatments attempted, Assistant B's use of "chronic idiopathic urticaria" provides a slightly more precise summary of the patient's situation. However, Assistant A's explicit mention of antihistamines and "other treatments" offers a more detailed account of the patient's treatment history.

Considering these factors, the decision is very close, but the slight edge in accuracy and specificity of the medical condition gives Assistant B a minor advantage. However, the difference is minimal, and both summaries effectively capture the essence of the patient's query.

**Final Verdict:** [[B]]
