# Supplementary material for: A Multiagent Summarization and Auto-Evaluation Framework for Medical Text: Development and Evaluation Study
Source: JMIR AI. 2025 Dec 16;4:e75932. doi: 10.2196/75932 (PMC12707800; doi:10.2196/75932)
Supplement: Multimedia Appendix 3 [file ai-v4-e75932-s003.docx]

## S1. Expert Participants’ Data Compilation Format

Each decision maker, the LLM or a human expert, as a judge selects the better summary from two generated summaries for each data point in a dataset. The decisions are compiled in a 2x2 array with *n* rows for *n* data points in the dataset, and *J* columns for each judge. Each column contains one of the 3 possible decision values D = {A, B, C}, indicating which summary is better or that both summaries are of the same quality (tie). Table 1 shows an example of the data compilation format.

Table S1: Data Compilation Format using a 2x2 Array. ‘A’, ‘B’, and ‘C represent summary A or B is better or both summaries are of same quality indicating a tie (C). The example shows a dataset with *n* datapoints or generated summaries and *J* judges, human and/or LLM.

|  | Judge 1 | Judge 2 | Judge 3 | Judge 4 |
| --- | --- | --- | --- | --- |
| Data 1 | A | A | … | C |
| Data 2 | B | B | B | A |
| ..... | ... | ... | … | ... |
| Data *n* | B | C | … | B |

## S1.1 Counting Decision Types (A, B, C)

For the *i* th data point in Table 1, we count the number of times decision type *d∈ D* was chosen by the judges using Eq. 1. *C_di_* indicates the number of judges in agreement with the decision *d∈D* for the data point *i.*

$$\begin{aligned} C_{di} = \sum_{j=1}^{J} 1\left( y_{ij}=d \right), \forall d\in D \#\left( 1 \right) \end{aligned}$$

Where:

- $J$: Total number of judges.
- $j$: Index of each judge.
- $i$: Index of each sample.
- $D$: Three decision values {A, B, C}.
- $d$: $\forall d\in D$.
- $C_{di}$: Number of judges who chose decision d for data point $i$.
- $y_{ij}$: The decision made by judge $j$ for data point $i$.
- $1(y_{ij}=d)$: Indicator function (1 if $y_{ij}$ is equal to $d$, 0 if $y_{ij}$ is not equal to $d$).

For example, for row 1 in Table 1, Eq. 1 will provide {2*_d=A_*, 0*_d=B_*, 1*_d=C_*} for each decision in {A, B, C}, which shows 2 judges agree with the decision that the 1^st^ summary is better than the 2^nd^ summary, 0 judges chose the 2^nd^ summary and 1 judge chose a tie.

## S1.2 Computing Agreement Scores for Judges

To compute the number of agreements among at least *m* judges out of *J*, we modify Eq. 1 to add a constraint as shown in Eq. 2.

$$\begin{aligned} {Agreement}_{d}^{\left( m \right)}= \sum_{i=1}^{n} 1\left( {max(C}_{i})\geq m \right), \forall d\in D\#\left( 2 \right) \end{aligned}$$

Where:

- $n$: Total number of samples.
- $i$: index of each sample (from $i$ to $n$).
- $D$: Three decision values {A, B, C}.
- $d$: $\forall d\in D$.
- $C_{i}$: ​ It is the vote count vector for sample 𝑖, containing the number of judges who selected each decision. It is constructed by collecting all $C_{di}$ values for that sample across all decisions $d\in D$.
- ${max(C}_{di})$: The largest number of votes for any decision in sample 𝑖.
- $1\left( {max(C}_{di})\geq m \right)$: An indictor function that is 1 if $\left( {max(C}_{i})\geq m \right)$, and 0 otherwise.

For example, for row 1 in Table 1, where the decisions made by the judges result in the vote count vector: $C_{i}$= {2*_d=A_*, 0*_d=B_*, 1*_d=C_*}. Given a threshold *m* = 2, we compute ${max(C}_{i})$=2. Since ${max(C}_{i})\geq m$, this sample satisfies the agreement condition and contributes a value of 1 to the overall agreement score. We then repeat this process for all remaining samples and aggregate the results to compute the total number of samples where at least 𝑚 judges agree on any single decision.

## S1.3 Computing Majority Decision of Judges

To compute the majority decision of judges for each data point in the dataset from *J* judges, we use Eq. 3 as follows.

$$\begin{aligned} {MD}_{i}= \left\{ \begin{aligned} \text{"}\text{C}\text{"}, if\max\left( C_{\left( d=A \right)i},C_{\left( d=B \right)i},C_{\left( d=C \right)i} \right)\leq\left( 0.5\cdot j \right) \\ \text{"}\text{C}\text{"}, if C_{\left( d=A \right)i}=C_{\left( d=B \right)i} \\ argmax\left( C_{\left( d=A \right)i},C_{\left( d=B \right)i},C_{\left( d=C \right)i} \right)=d), otherwise, \forall d\in D \end{aligned} \right.\#\left( 3 \right) \end{aligned}$$

Where:

- $n$: Total number of samples.
- $i$: index of each sample (from $i$ to $n$).
- $D$: Three decision values {A, B, C}.
- $d$: $\forall d\in D$.
- $\max\left( C_{\left( d=A \right)i},C_{\left( d=B \right)i},C_{\left( d=C \right)i} \right)$: The maximum number of judges in agreement in selecting decision *A*, *B*, or *C* for sample *i*.
- *J*: Total number of judges.
- $argmax\left( C_{(d=A)i},C_{\left( d=B \right)i},C_{\left( d=C \right)i} \right)$: Decision *d* that received the highest vote for sample $i$.
- *MD_i_*: Majority decision of judges
  - Eq. 3 line 1: If no single decision (*A*, *B*, or *C*) receives more than half of the total votes, the result is considered a tie.
  - Eq. 3 line 2: If decision *A* and *B* receive the same number of votes, the result is considered a tie.
  - Eq. 3 line 3: If neither of the above applies, the option with the most votes wins.

For example, for row 2 in Table 1, *MD_i_* will provide *B* as the majority decision based on Eq. 3 line 3.

## S1.4 Compute Agreement Score between LLM and Human Expert

To compute the agreement score between two groups of judges, LLMs and humans, in choosing the best summary, we compare the corresponding majority decisions ${MD}_{i}^{L}$and ${MD}_{i}^{H}$of the group of LLMs and human judges.

$$\begin{aligned} {Agreement_{Score}}_{i}=\sum_{i=1}^{n} 1\left( {MD}_{i}^{L}={MD}_{i}^{H} \right) \#\left( 4 \right) \end{aligned}$$

Where:

- $n$: Total number of samples.
- $i$: index of each sample (from $i$ to $n$).
- ${MD}_{i}^{L}$: Majority decision of LLMs for data point $i$.
- ${MD}_{i}^{H}$: Majority decision of human experts for data point $i$.
- $1({MD}_{i}^{L}={MD}_{i}^{H})$: Indicator function that returns 1 if ${MD}_{i}^{L}$ and ${MD}_{i}^{H}$agree with each other on sample $i$, else 0.

To compute the agreement score between LLMs and human experts, we compare their majority decisions (MDᵢᴸ and MDᵢᴴ) for each sample i and count how often they match. The score is calculated by aggregating the indicator function $1\left( {MD}_{i}^{L}={MD}_{i}^{H} \right)$ across all samples, providing a simple measure of alignment between the two groups.

Table S2. Average AR between GPT-4 judge with Prompt-EG and each of the 4 experts in preferred summary selection for 5 datasets. The calculation details are provided in Section S1.4.

| **Human Experts** | **E1** | **E2** | **E3** | **E4** |
| --- | --- | --- | --- | --- |
| Average AR between GPT-4 and Human Expert (%) | 42.18 | 43.79 | 50.54 | 36.65 |
