# Supplementary material for: A Multiagent Summarization and Auto-Evaluation Framework for Medical Text: Development and Evaluation Study
Source: JMIR AI. 2025 Dec 16;4:e75932. doi: 10.2196/75932 (PMC12707800; doi:10.2196/75932)
Supplement: Multimedia Appendix 4 [file ai-v4-e75932-s004.docx]

# Appendix 4: Examples of GPT-4’s Evaluation Behavior

This document presents examples that highlight key aspects of GPT-4’s behavior as an evaluator in pairwise summary selection tasks. Figure A4.1 and Figure A4.2 demonstrate how GPT-4 may exhibit position bias, showing consistent preference for the same summary content regardless of its position. Figure 3 showcases GPT-4’s capability to detect hallucinated content in summaries and justify its selection.

Figure A4.1. Example of GPT-4’s judgment in selecting the better summary from a pair generated by two different LLMs. The highlighted section indicates the summary preferred by the GPT-4 judge.


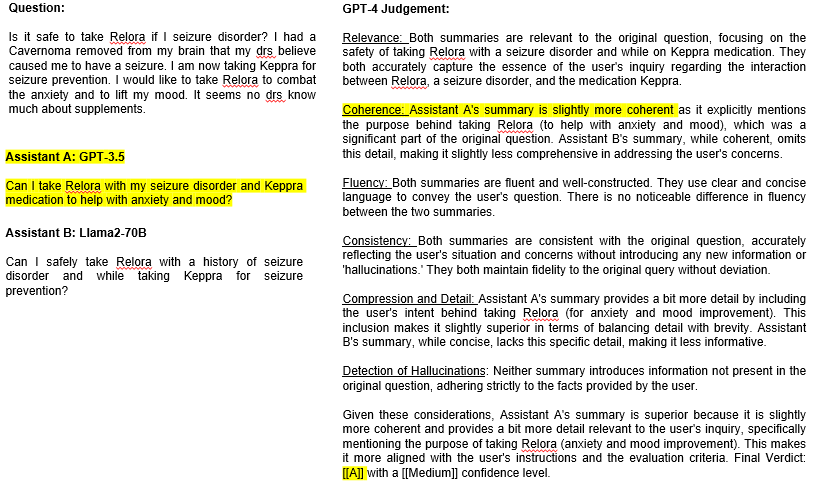

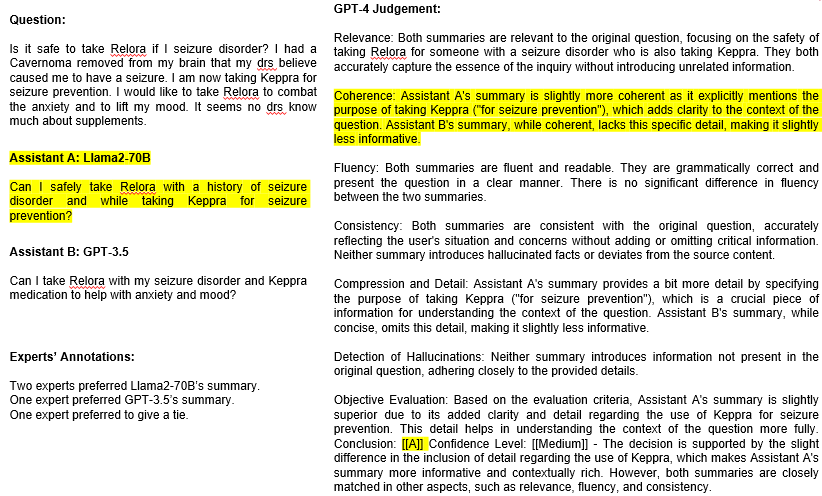


Figure A4.2. Example of GPT-4’s judgment when the input order of the two LLM-generated summaries from Figure A4.1 is swapped. In both Figures 1 and 2, GPT-4 selects Assistant A as the preferred summary. However, since the summaries are generated by different LLMs in each position, this illustrates the issue of position bias in GPT-4’s evaluation.


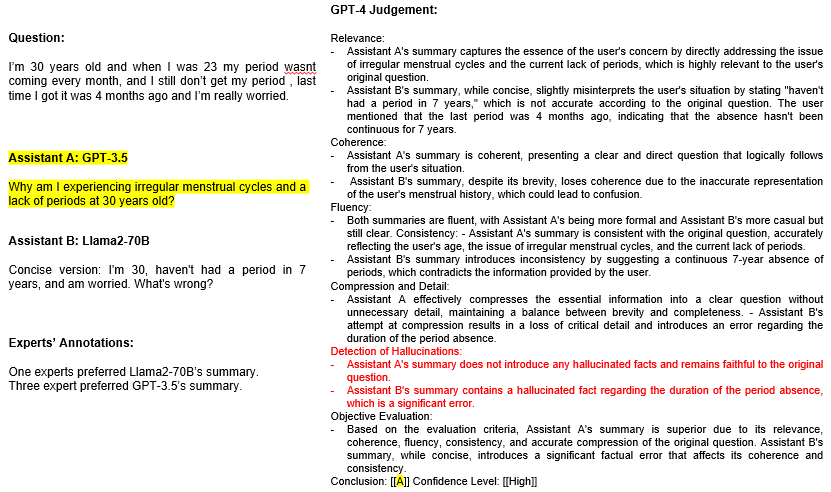


Figure A3. Example of a hallucination identified directly by GPT-4. The red highlighted section indicates GPT-4’s explanation of the hallucinated content in the summary pair. The yellow highlighted section marks the summary preferred by GPT-4, which also aligns with the majority decision of human experts.
