# Supplementary material for: A Multiagent Summarization and Auto-Evaluation Framework for Medical Text: Development and Evaluation Study
Source: JMIR AI. 2025 Dec 16;4:e75932. doi: 10.2196/75932 (PMC12707800; doi:10.2196/75932)
Supplement: Multimedia Appendix 5 [file ai-v4-e75932-s005.docx]

# Appendix 5: Factually Incorrect Hallucination Detection

We define Factually Incorrect Hallucination (FIH) as a model output that contradicts with the source text or introduces claims inconsistent with established medical knowledge. We use our Prompt-EG as GPT-4 (Broad) to select the best summary and detect hallucinated summaries. Prompt-EG was originally designed to evaluate multiple aspects of summary quality (coherence, consistency, fluency, and relevance), but it also demonstrates the ability to identify hallucinations that arise during the reasoning process leading to the final evaluation. For comparison, we define a more focused hallucination detection only prompt as GPT-4 (Focused) (see Table A5.3). Both these prompts generate a binary output with respect to hallucination detection. Four human annotators were recruited to collaboratively evaluate the summaries and model predictions and reach a consensus in identifying summaries with FIH. Results are reported as macro-averaged values of FIH and non-FIH cases in terms of accuracy, precision, sensitivity, specificity, and F1 score. Confidence intervals are estimated using 10,000 bootstrap resamples based on 100 question summarization and 50 dialog summarization samples with replacement. Due to time constraints, we limited this analysis to the question summarization and dialog summarization tasks, as answer summarization requires substantially more time and effort for human evaluation. In Table A5.1 and Table 5,2, we present the human evaluation results of detected hallucination cases by GPT-4 under two prompt conditions.

**Table A5.1. The number of FIH cases detected by the different methods out of the total number of data points.**

| **Dataset** | **Question Summarization** | **Dialog Summarization** |
| --- | --- | --- |
| GPT-4 (Broad) | 3 / 100 | 2 / 50 |
| GPT-4 (Focused) | 14 / 100 | 8 / 50 |
| Human | 10 / 100 | 3 / 50 |

**Table A5.2. Human evaluation of automatic FIH detection by LLMs presented in percentage. The upper row in each cell reports the evaluation result, and the lower row shows the 95% confidence interval.**

| **Dataset** | **Model** | **Accuracy (%)** | **Precision (%)** | **Sensitivity (%)** | **Specificity (%)** | **F1 Score (%)** |
| --- | --- | --- | --- | --- | --- | --- |
| **Question Summari-zation** | **GPT-4 (Broad)** | 93.0 (88.0–97.0) | 96.4 (46.5-98.5) | 65.0 (50.0, 81.2) | 100 (100– 100) | 71.2 (50.0-87.7 |
|  | **GPT-4 (Focused)** | 86.0 (79.0-92.0) | 65.0 (52.3-78.8) | 70 (53.4-86.7) | 90.0 (83.3-95.6) | 66.9 (52.0,79.6) |
| **Dialog Summari-zation** | **GPT-4 (Broad)** | 98.0  (94.0–100) | 99.0  (48.0–100) | 83.3 (50.0–100) | 100  (100–100) | 89.5 (49.0–100) |
|  | **GPT-4 (Focused)** | 90.0  (80.0–98.0) | 68.8  (50.0–87.5) | 94.7 (45.0 –98.9) | 89.4 (80.0–97.8) | 74.5  (47.4–92.3) |

Table A5.1 summarizes the number of FIH cases detected by each model and by human evaluators. GPT-4 (Focused) identified more hallucination cases than GPT-4 (Broad), and its total count was closer to that of the human evaluation. This suggests that the focused prompt is more sensitive and likely to flag potential hallucinations that the broad prompt overlooks. However, a higher count does not necessarily indicate higher accuracy, as some of these detections may include false positives.

The results of this experiment offer a crucial insight: prompt specificity is the primary driver for the quality of FIH detection. As shown in Table A5.2, transitioning GPT-4 from a broad, multi-aspect prompt to a focused, single-task prompt revealed a trade-off between sensitivity and other performance metrics. The focused prompt improved sensitivity, increasing from 65.0 % (95 % CI 50.0–81.2) to 70.0 % (95 % CI 53.4–86.7) for question summarization and from 83.3 % (95 % CI 50.0–100.0) to 94.7 % (95 % CI 45.0–98.9) for dialog summarization. This indicates a reduction in false negatives, meaning that fewer FIH hallucinations went undetected. However, this improvement came at the expense of precision, specificity, and overall accuracy, all of which declined relative to the broader prompt condition.

Even with this limitation, GPT-4 (Broad) showed the most balanced and reliable performance. In the dialog summarization task, it reached an F1 score of 89.5 % (95 % CI 49.0–100.0) and perfect specificity (100 %). Compared with GPT-4 (Focused), the broad version achieved higher precision and specificity, meaning it was better at avoiding false positives. The focused version, however, showed slightly higher sensitivity, detecting more FIHs.

To investigate the results, we show two examples from the outputs of GPT-4 (Broad and Focused). In Example 1, LLM Assistant A misinterprets the phrase “when a person starts halt sildenafil.” The intended meaning is “when a person begins to stop using sildenafil,” but LLM Assistant A hallucinates a different interpretation: “starting or stopping sildenafil.” GPT-4 (Broad) fails to detect this FIH. In contrast, GPT-4 (Focused) correctly identifies that the original input does not convey the concept of starting sildenafil and appropriately flags the assistant's error.

Example 2 illustrates the case that GPT-4 (Broad and Focused) failed to detect. In the source text, the patient mentioned reading about type 1 diabetes but did not state whether their friend actually had the condition. Both generated summaries, however, introduced this unsupported information.

In Example 3, the model replaced an abbreviated term with its full form, which was not explicitly stated in the source but is factually correct and contextually aligned. Both human annotators and LLM judges did not classify such cases as FIH, as long as the expanded term was a correct and unambiguous interpretation of the original abbreviation

**Table A5.3. GPT-4 Focused prompt for only hallucination detection for question and dialog summarization tasks.**

| **Dataset** | **Prompt-baseline for the Evaluation Layer** |
| --- | --- |
| Consumer Health Question Summarization | In this task, you are required to act as a medical expert and determine whether either of the two summaries contains hallucinated information. A hallucination is defined as any content in the summary that is not supported by or grounded in the original user question.  Please follow these instructions:  Step 1: Understand the Source  * Read the original user question carefully. Identify the key facts, concerns, symptoms, or topics mentioned in the question. This forms the ground truth that summaries should stay faithful to.  Step 2: Analyze Summary A  * Read Assistant A's summary.  * Go sentence by sentence.  * For each sentence, ask:  - Is this information directly supported by the original question?  - Does this sentence introduce any new facts or claims that are not implied or stated in the user question?  * If any such unsupported information is found, mark this summary as containing a hallucination.  * Provide a brief reason explaining your decision.  Step 3: Analyze Summary B  * Repeat the same process as in Step 2 for Assistant B's summary.  Important Notes:  * Do not evaluate fluency, coherence, relevance, or other language quality aspects.  * Only focus on whether hallucinated (unsupported) content is present in either summary.  Output Format  * After reviewing both summaries, output your judgment in the following specific format:  Assistant A: [[H]] or [[no_H]]  Reason: <short explanation based on your step-by-step reasoning>  Assistant B: [[H]] or [[no_H]]  Reason: <short explanation based on your step-by-step reasoning> |
| Dialog Summarization | In this task, you are required to act as a medical expert and determine whether either of the two summaries contains hallucinated information. A hallucination is defined as any content in the summary that is not supported by or grounded in the dialog transcript. This includes any information not mentioned or inferable from the patient–doctor conversation.  Please follow these instructions:  Step 1: Understand the Source  * Carefully read the full dialog transcript between the patient and the medical professional.  * This dialog serves as the ground truth for evaluating factual consistency in each summary.  Step 2: Analyze Summary A  * Read Assistant A's summary.  * Go sentence by sentence.  * For each sentence, ask:  - Is this information directly supported by the dialog?  - Does this sentence introduce any new facts or claims that are not implied or stated in the dialog?  * If any such unsupported information is found, mark this summary as containing a hallucination.  * Provide a brief reason explaining your decision.  Step 3: Analyze Summary B  * Repeat the same process as in Step 2 for Assistant B's summary.  Important Notes:  * Do not evaluate fluency, coherence, relevance, or other language quality aspects.  * Only focus on whether hallucinated (unsupported) content is present in either summary.  Output Format  * After reviewing both summaries, output your judgment in the following specific format:  Assistant A: [[H]] or [[no_H]]  Reason: <short explanation based on your step-by-step reasoning>  Assistant B: [[H]] or [[no_H]]  Reason: <short explanation based on your step-by-step reasoning> |

# Example 1

## Source for summarization:

SUBJECT: time of results. MESSAGE: When a person start halt sildenafil...How long before their breathing, shortness of breath, &fatigue issues go back to normal?

## Assistant A’s summary:

How long does it take for breathing, shortness of breath, and fatigue to improve after starting or stopping sildenafil?

## Assistant B’s summary:

How long after stopping sildenafil do breathing, shortness of breath, and fatigue issues return to normal?

## GPT-4 (Broad) Factually Incorrect Hallucination Detection:

**Relevance:** Both summaries are relevant to the original question, focusing on the key aspect of how long it takes for breathing, shortness of breath, and fatigue issues to normalize after halting sildenafil. Neither summary introduces irrelevant information.

**Coherence:** Assistant B's summary is slightly more coherent as it directly addresses the question's main concern in a concise manner. Assistant A's summary, while still coherent, introduces ambiguity by mentioning "starting or stopping" sildenafil, which could confuse readers about the direction of the inquiry (the original question specifically asks about stopping the medication).

**Fluency:** Both summaries are fluent and well-constructed. Assistant A and B both use clear and understandable language to convey the message.

**Consistency:** Assistant B's summary is more consistent with the original question as it directly mirrors the inquiry about the effects after stopping sildenafil. Assistant A's summary introduces a potential inconsistency by including the effects of starting sildenafil, which was not part of the original question.

**Compression and Detail:** Both summaries effectively compress the essential information from the original inquiry, maintaining a balance between detail and brevity. However, Assistant B's summary does so with slightly better alignment to the original question's focus.

**Detection of Hallucinations:** Neither summary introduces hallucinated facts or information not present in the original question.

**Objective Evaluation:** Based on the evaluation criteria, Assistant B's summary is superior due to its direct alignment with the original question, coherence, and consistency. Assistant A's inclusion of starting sildenafil introduces a slight deviation from the original inquiry, affecting its relevance and consistency.

Final Verdict: [[B]] with a [[Medium]] confidence level. The decision is supported by noticeable differences in coherence and consistency, with Assistant B's summary more accurately reflecting the original question's intent. However, the close similarity in fluency and relevance between the summaries prevents a high confidence rating.

## GPT-4 (Focused) Factually Incorrect Hallucination Detection:

Assistant A: [[H]]

Reason: The summary introduces the concept of "starting" sildenafil, which is not mentioned or implied in the original question. The question only asks about the effects after stopping the medication.

Assistant B: [[no_H]]

Reason: This summary accurately reflects the original question's focus on the timeframe for improvement in symptoms after stopping sildenafil, without introducing any new, unsupported information.

# Example 2

## Source for Summarization:

SUBJECT: diabetes

MESSAGE: It was really helpful after reading about type 1 diabetes but would like some more suggestions from you .my friend is 35 year old and its been 1 year of his diabetes n his sugar level is around 100 or 120 OR SO.. each time he tests his sugar level. is there ANY COMPLICATIONS? HE ALSO GO FOR EXERCISES AND BLOOD TEST EVERY 2 OR 3 MONTHS N TAKES A PROPER DIET LIKE GREEN JUICE,OLIVE OIL AND ALL. WAITING FOR YOUR FEED BACK. THANK YOU.

## Assistant A’s Summary:

Could you provide suggestions for managing type 1 diabetes in a 35-year-old who has had the condition for a year, with sugar levels around 100-120, exercises regularly, goes for blood tests every 2-3 months, and follows a proper diet? Any potential complications to be aware of? Thank you.

## Assistant B’s Summary:

What are the potential complications for a 35-year-old with type 1 diabetes, with a consistent blood sugar level of 100-120, who exercises regularly and follows a healthy diet?

## GPT-4 (Broad) Factually Incorrect Hallucination Detection:

**Relevance:** Both summaries accurately capture the essence of the user's request, focusing on the management of type 1 diabetes in a 35-year-old, the individual's current health practices, and the inquiry about potential complications. Assistant A provides a more detailed summary by explicitly mentioning the frequency of blood tests and specific dietary habits, which aligns closely with the user's original message.

**Coherence:** Assistant A's summary is more coherent, as it systematically lists the patient's practices (exercise, blood tests, diet) and then asks about management suggestions and potential complications, mirroring the structure of the original question. Assistant B's summary, while coherent, is more concise and slightly less structured in comparison.

**Fluency:** Both summaries are fluent, but Assistant A's use of a detailed and structured sentence enhances the readability and understanding of the user's situation and questions.

**Consistency:** Assistant A's summary is more consistent with the original question, capturing all the key elements mentioned by the user, including the specific details about diet and the frequency of blood tests. Assistant B's summary, while accurate, omits these details, which could be considered relevant for understanding the full context of the user's inquiry.

**Compression and Detail:** Assistant A's summary balances detail and brevity effectively, providing a comprehensive overview without unnecessary elaboration. Assistant B's summary, though concise, lacks some of the specific details that might be important for a complete understanding of the user's situation.

**Detection of Hallucinations:** Neither summary introduces information not present in the original question, maintaining a high level of factual accuracy.

Based on these criteria, Assistant A's summary is superior due to its greater detail, coherence, and consistency with the original question, without sacrificing fluency or relevance.

**Conclusion:** [[A]]

**Confidence Level:** [[High]]

## GPT-4 (Focused) Factually Incorrect Hallucination Detection:

Assistant A: [[no_H]]

Reason: Assistant A's summary accurately reflects the original question's content without adding any new, unsupported information. It directly addresses the user's request for suggestions on managing type 1 diabetes, mentions the friend's age, duration of diabetes, sugar levels, exercise routine, blood test frequency, and diet, all of which are mentioned in the original question.

Assistant B: [[no_H]]

Reason: Assistant B's summary also stays true to the original question, focusing on the potential complications for a person with the described health profile. It does not introduce any new facts or claims not present in the user's question.

# Example 3

## Source for Summarization:

SUBJECT: Sle

MESSAGE: How much level of feritin is responsible for SLE? Adult still's disease is related with Sle? How we differ them?

## Assistant A’s Summary:

What is the relationship between ferritin levels and SLE? How can Adult Still's disease be distinguished from SLE?

## Assistant B’s Summary:

What is the appropriate ferritin level for diagnosing Systemic Lupus Erythematosus (SLE), and how does it differ from Adult Still's disease?

## GPT-4 (Broad) Factually Incorrect Hallucination Detection:

**Relevance:** Both summaries aim to address the user's questions regarding the relationship between ferritin levels and SLE, and the differentiation between Adult Still's disease and SLE. Assistant A directly mirrors the user's questions, maintaining a high level of relevance. Assistant B rephrases the question about ferritin levels in terms of "appropriate ferritin level for diagnosing SLE," which slightly deviates from the user's original query about the level of ferritin responsible for SLE. This could imply a diagnostic criterion rather than a pathophysiological relationship, which is not exactly what the user asked.

**Coherence:** Assistant A's summary is straightforward and coherent, presenting the questions in a clear, concise manner. Assistant B's summary, while also coherent, introduces a slight ambiguity by suggesting the ferritin level is used for diagnosing SLE, which may not align perfectly with the user's intent.

**Fluency:** Both summaries are fluent, with Assistant A using a direct approach to restate the user's questions, and Assistant B employing a slightly more formal rephrasing. There are no issues with grammar or readability in either summary.

**Consistency:** Assistant A's summary is consistent with the original question, accurately reflecting the user's inquiries without introducing new information or misinterpreting the questions. Assistant B's summary, while still relevant, introduces a minor inconsistency by framing the ferritin level as a diagnostic criterion for SLE, which is not explicitly stated in the user's question.

**Compression and Detail:** Assistant A maintains a high level of detail from the original question without unnecessary elaboration, effectively compressing the user's inquiry into a concise summary. Assistant B also compresses the information but slightly alters the focus regarding ferritin levels, which could lead to a misunderstanding of the user's original intent.

**Detection of Hallucinations:** Neither summary introduces hallucinated facts; both stay true to the content of the original question.

Given these considerations, Assistant A's summary is superior because it more accurately and directly reflects the user's original questions without introducing potential misunderstandings. Assistant B's slight deviation in framing the question about ferritin levels introduces a minor inconsistency with the user's intent.

[[A]] - Assistant A's summary is superior.

[[High]] - The decision is made with a clear and definitive assessment, where Assistant A's summary distinctly outperforms Assistant B's summary in terms of relevance and consistency, leading to minimal doubt about the conclusion.

## GPT-4 (Focused) Factually Incorrect Hallucination Detection:

Assistant A: [[no_H]]

Reason: Assistant A accurately paraphrases the user's questions without adding any new information or making unsupported claims. It directly addresses the user's inquiries about the relationship between ferritin levels and SLE and how to distinguish Adult Still's disease from SLE.

Assistant B: [[no_H]]

Reason: Assistant B also accurately reformulates the user's questions, focusing on the appropriate ferritin level for diagnosing SLE and the differentiation between SLE and Adult Still's disease. It does not introduce any new or unsupported information.
